# Dipyridamole Acts as Clinical Ferroptosis Inhibitor to Prevent from Tissue Injury

**DOI:** 10.1002/advs.202500566

**Published:** 2025-05-14

**Authors:** Xiao Zhuang, Shuang Shi, Shuo Liu, Yaqiong Jiao, Bin Huang, Yinghong Yang, Li Yang, Xinquan Yang, Hui Wang, Chunhui Liang, Dandan Song, Huaxiang Yu, Dan Zou, Qi Sun, Shu Yang, Chengqian Yin, Jian Li, Yiming Liu, Junxia Min, Fudi Wang, Yong Nian, Lutao Du, Bo Chu

**Affiliations:** ^1^ Department of Cell Biology School of Basic Medical Sciences Cheeloo College of Medicine Shandong University Jinan 250012 China; ^2^ Department of Clinical Laboratory The Second Hospital of Shandong University Jinan 250012 China; ^3^ Department of Geriatric Medicine Qilu Hospital of Shandong University Jinan 250012 China; ^4^ Department of General Practice Qilu Hospital of Shandong University Jinan 250012 China; ^5^ Institute for Cancer Research Shenzhen Bay Laboratory Shenzhen 518107 China; ^6^ Department of Respiratory and Critical Care Medicine Zhengzhou University People's Hospital Henan Provincial People's Hospital Zhengzhou 450003 China; ^7^ The First Affiliated Hospital Institute of Translational Medicine Zhejiang University School of Medicine Hangzhou 310058 China; ^8^ Department of Emergency Medicine and Chest Pain Center Qilu Hospital of Shandong University Jinan 250012 China; ^9^ Department of Biochemistry and Molecular Biology Shandong University School of Medicine Jinan 250012 China; ^10^ College of Pharmacy Nanjing drum tower hospital Nanjing University of Chinese Medicine Nanjing 210023 China; ^11^ Department of Neurology Qilu Hospital of Shandong University Jinan 250012 China; ^12^ The First Affiliated Hospital The Second Affiliated Hospital Institute of Translational Medicine School of Public Health State Key Laboratory of Experimental Hematology Zhejiang University School of Medicine Hangzhou 310058 China; ^13^ Department of Clinical Laboratory Qilu Hospital of Shandong University Jinan 250012 China; ^14^ Shandong Provincial Key Laboratory of Innovation Technology in Laboratory Medicine Jinan 250012 China

**Keywords:** dipyridamole, ferroptosis, SLC7A11, tissue injury

## Abstract

Ferroptosis is a newly identified cell death triggered by iron‐induced lipid peroxidation. Numerous studies reveal that ferroptosis participates in multiple types of tissue injury including ischaemia–reperfusion (I/R) injury and doxorubicin (Dox)‐induced damage. Targeting ferroptosis is a promising approach for disease treatment as the blockade of ferroptosis efficiently alleviates the symptoms. However, no known ferroptosis inhibitors have been used for clinical treatment. Although certain clinical compounds act as ferroptosis inhibitors in vitro, whether these drugs cure tissue injury by suppressing ferroptosis is little known. Here, by screening a large panel of drugs used in the clinic, it is identified that dipyridamole significantly attenuates Dox or I/R‐induced cardiac injury. Moreover, dipyridamole can achieve a good therapeutic effect on  liver and kidney injury. Mechanistically, dipyridamole‐mediated ferroptosis inhibition is strictly dependent on solute carrier family 7 member 11 (SLC7A11). Dipyridamole down‐regulates the expression of ring finger protein 126 (RNF126), which is an E3 ligase to ubiquitinate SLC7A11 for proteasome degradation. Deficiency of SLC7A11 largely blocks the protective role of dipyridamole in vitro and in vivo. Together, the findings uncover that dipyridamole acts as a clinical compound to alleviate organ injury via suppressing ferroptosis, providing novel insights into the clinical therapy for ferroptosis‐related tissue damage.

## Graphical abstract

Dipyridamole acts as a clinically used compound to suppress ferroptosis. Dipyridamole down‐regulates the expression of RNF126, which is an E3 ligase to ubiquitinate SLC7A11 for proteasome degradation. The deficiency of SLC7A11 largely abolishes the protective role of dipyridamole both in vitro and in vivo.

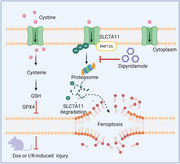

## 1 Introduction

Ferroptosis is a new programmed cell death distinct from apoptosis and necrosis. The occurrence of ferroptosis is induced by accumulated peroxidized phospholipids in an iron‐dependent manner.^[^ ^1^ ^]^ Polyunsaturated fatty acids (PUFAs), the major substrates of lipid peroxidation, are integrated into membrane phospholipids (PLs) to form PLs‐PUFA via Acyl‐CoA synthetase long‐chain family member 4 (ACSL4) and lysophosphatidylcholine acyltransferase 3.^[^ ^2^, ^3^ ^]^ The two isoforms of PUFA contain one (PL‐PUFA_1_) and two polyunsaturated fatty acyl tails (PLs‐PUFA_2_), respectively. Whereas PL‐PUFA_1_ has been considered as the major factor to drive ferroptosis, most recent research declares that PL‐PUFA_2_ renders cells more sensitive to ferroptosis.^[^ ^4^ ^]^ Non‐enzymatic or lipoxygenase‐stimulated peroxidation of PLs‐PUFA induces ferroptotic cell death.^[^ ^5^, ^6^, ^7^ ^]^ Excess peroxidized lipids could be eliminated by multiple defense response pathways including glutathione peroxidase 4 (GPX4), ferroptosis suppressor protein 1 (FSP1), GTP cyclohydrolase 1 (GCH1), dihydroorotate dehydrogenase (DHODH), tryptophan and cholesterol metabolism systems and membrane bound O acyltransferase domain containing 1/2.^[^ ^8^, ^9^, ^10^, ^11^, ^12^, ^13^, ^14^, ^15^, ^16^ ^]^

Emerging evidence indicates that ferroptosis is implicated in ischaemia–reperfusion (I/R), metabolism disorder‐ and drugs‐induced tissue injury. For instance, induction of ferroptosis in certain organs such as the liver or kidney by specifically genetic depletion of GPX4 or I/R surgical operation.^[^ ^16^, ^17^, ^18^ ^]^ High fat or methionine‐choline deficient diet‐induced metabolism disorder triggers ferroptosis in liver and non‐alcoholic fatty liver disease.^[^ ^19^, ^20^ ^]^ FerroTerminator1 effectively reverses liver injury across multiple MASH models without notable toxic side effects compared with clinically approved iron chelators^[^ ^21^ ^]^; Using pharmacological ferrostatin‐1 (Fer‐1) methods substantially ameliorated I/R injury in the heart^[^ ^22^ ^]^; Ferroptosis inhibitors, such as iron chelation and Fer‐1, ameliorate heart failure induced by acute and chronic I/R in mice ^[^ ^23^, ^24^ ^]^; Using a combined small molecule inhibitor (Nec‐1f) that simultaneously targets receptor interacting protein kinase 1 and induces ferroptosis in freshly isolated primary kidney tubules, and in mouse models of cardiac transplantation and acute kidney injury, they improved survival in models of I/R injury.^[^ ^25^ ^]^ The indiscriminate use of the anti‐cancer drug doxorubicin (Dox) brings out severe cardiac injury.^[^ ^23^, ^26^ ^]^ All these symptoms could be efficiently alleviated by classical ferroptosis inhibitors Fer‐1 and liprostatin‐1 (Lipro‐1), indicating that targeting ferroptosis should be a promising therapeutic approach for multiple types of organ damage. However, until now, no experimental ferroptosis inhibitors are used for clinical treatment. Certain well‐known ferroptosis inhibitors including Lipro‐1 and deferoxamine are still undergoing pre‐clinical or clinical trials limited to drug stability, efficiency, or toxicity in vivo. Numerous studies revealed that certain clinical drugs have been identified as ferroptosis inhibitors.^[^ ^23^, ^27^, ^28^, ^29^, ^30^ ^]^ However, it should be noted that few clinical compounds as ferroptosis inhibitor were utilized in the in vivo experiments to validate the role in defending against ferroptosis‐induced tissue injury. Considering the pivotal role of ferroptosis in tissue injury, it is urgent to explore whether clinically approved compounds act as potent ferroptosis suppressors to cure tissue injury.

In the present study, we show that dipyridamole exhibits a robust effect to protect against ferroptosis via screening a clinical drug library. Administration of dipyridamole markedly attenuates I/R‐ or Dox‐induced tissue injury before or after insult in a preclinical mouse model. Notably, the protective effect of dipyridamole is diminished in solute carrier family 7 member 11 knockout (SLC7A11 KO) mice, suggesting that dipyridamole‐mediated ferroptosis inhibition and tissue protection are dependent on SLC7A11. Further, co‐immunoprecipitation (Co‐IP) experiments elucidate that dipyridamole stabilizes SLC7A11 via downregulation of E3 ligase ring finger protein 126 (RNF126). In conclusion, our study suggests that dipyridamole may be a suitable clinical compound for therapy of ferroptosis‐induced tissue injury.

## 2 Results

### 2.1 Identification of Candidate Clinical Ferroptosis Inhibitors

**Figure 1:**
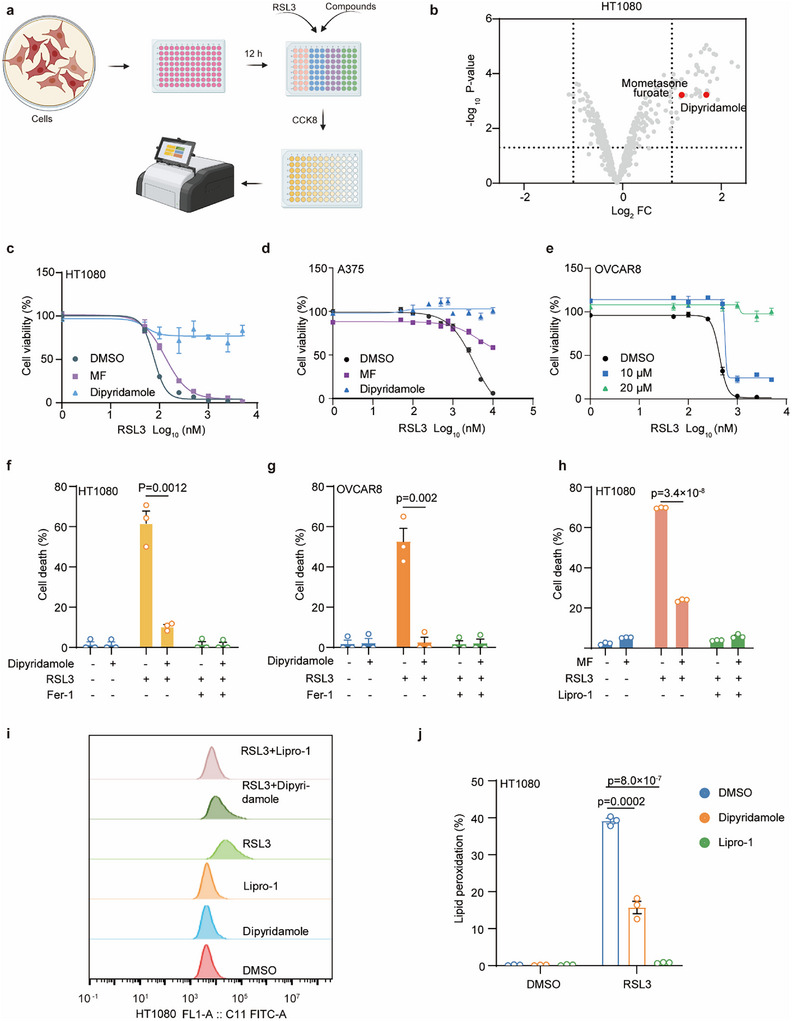
Identification of candidate clinical ferroptosis inhibitors. a) Schematic of identification of potential ferroptosis inhibitors for disease treatment, using HT1080 cells pretreated with clinical drugs (10 µm) followed by RSL3 treatment for 24 h. b) Volcano plots showing the top hits of clinical drug screen in HT1080 cells. c,d) Dose‐dependent toxicity of RSL3‐induced cell death of HT1080 (c) and A375 cells (d) after pre‐treatment with DMSO, dipyridamole (10 µm), or MF (10 µm) for 12 h. Cell viability was measured 12 h post‐treatment after using CCK8. e) Dose‐dependent toxicity of RSL3‐induced cell death in OVCAR8 cells after pre‐treatment with dipyridamole (10 and 20 µm) for 12 h. Cell viability was assessed 12 h post‐treatment after using CCK8. f,g) Cell death measurement of HT1080 (f) and OVCAR8 (g) cells treated with RSL3 (500 nm), dipyridamole (10 µm), Fer‐1 (4 µm) for 6 h. Dead cells were labeled with SYTOX Green. h) Cell death measurement of HT1080 cells treated with RSL3 (500 nm), MF (10 µm) and Lipro‐1(4 µm) for 6 h. Dead cells were labeled with SYTOX Green. i,j) BODIPY 581/591 C11 staining of lipid peroxidation in HT1080 cells treated with RSL3 (250 nm), Lipro‐1(4 µm) or dipyridamole (10 µm) for 4 h.  Data and error bars are mean ± SEM, *n*  =  3 biologically independent experiments in b–h and j. All *P* values were calculated using a two‐tailed, unpaired Student's *t*‐test.

Several categories of ferroptosis inhibitors have been developed for experimental action. However, all these compounds have not yet received approval from the Food and Drug Administration for clinical application. We, therefore, tested whether clinical drugs could act as potent ferroptosis inhibitors for disease treatment. Through the screening of a panel of commonly used drugs in clinical settings, we discovered that dipyridamole significantly diminished the occurrence of cell death through ferroptosis in human osteosarcoma cell line HT1080, human renal carcinoma cell line 786‐O and human cardiomyocyte cell line AC16 (**Figure** **1**a,b; Figure S1a,b, Supporting information). As shown in Figure 1c, ferroptotic cell death was induced in HT1080 cells by ferroptosis inducer RSL3. Administration of dipyridamole remarkably defended cells against RSL3‐induced ferroptosis in a dose‐dependent manner. Similar phenotypes were observed in human melanoma A375 and ovarian cancer OVCAR8 (Figure 1d,e). These results indicated that the protective effect of dipyridamole is a common phenomenon in various types of cell lines.

Additionally, we employed a cell death assay and observed that dipyridamole, similar to Fer‐1, specifically blocks ferroptotic cell death (Figure 1f,g; Figure S1c, Supporting information). To confirm that the observed cell death was indeed due to ferroptosis, we utilized the autophagy inhibitor 3‐Methyladenine (3‐MA), the necrosis inhibitor necrostatin‐1(Nec‐1), and the apoptosis inhibitor Z‐VAD‐FMK(Z‐VAD). The results indicated that only the ferroptosis inhibitor Fer‐1, as well as dipyridamole, could rescue cell death induced by RSL3 (Figure S1d, Supporting information). Furthermore, we induced ferroptotic cell death through alternative methods such as cystine/cysteine starvation, erastin, ML162, and ML210. These findings revealed that dipyridamole is a potent ferroptosis suppressor to protect against various ferroptosis inducers‐mediated cell death (Figure S1e–h, Supporting information).

Notably, we also observed that mometasone furoate (MF), which belongs to glucocorticoid, exhibited a protective effect to a lesser extent compared to dipyridamole (Figure 1b–d,h; Figure S1i, Supporting information). Dipyridamole has been used for ischemic heart disease, which is tightly related to ferroptosis. Furthermore, considering the effect of anti‐ferroptosis and the multiple side effects of long‐term abuse of glucocorticoid, we therefore focused our attention on the role of dipyridamole. Lipid peroxidation is a well‐known characteristic of ferroptosis and serves as a driving force for this type of cell death. To assess intracellular levels of lipid peroxidation, we employed the BODIPY 581/591 C11 fluorescence dye in conjunction with flow cytometry analysis. We observed a dramatic increase in lipid peroxidation in the cells treated with RSL3. As anticipated, both Lipro‐1 and dipyridamole effectively alleviated lipid peroxidation (Figure 1i,j). Overall, these findings highlight the essential role of dipyridamole in the regulation of ferroptosis.

### 2.2 Dipyridamole Inhibits Ferroptotic Cell Death in Myocardium Cells

**Figure 2:**
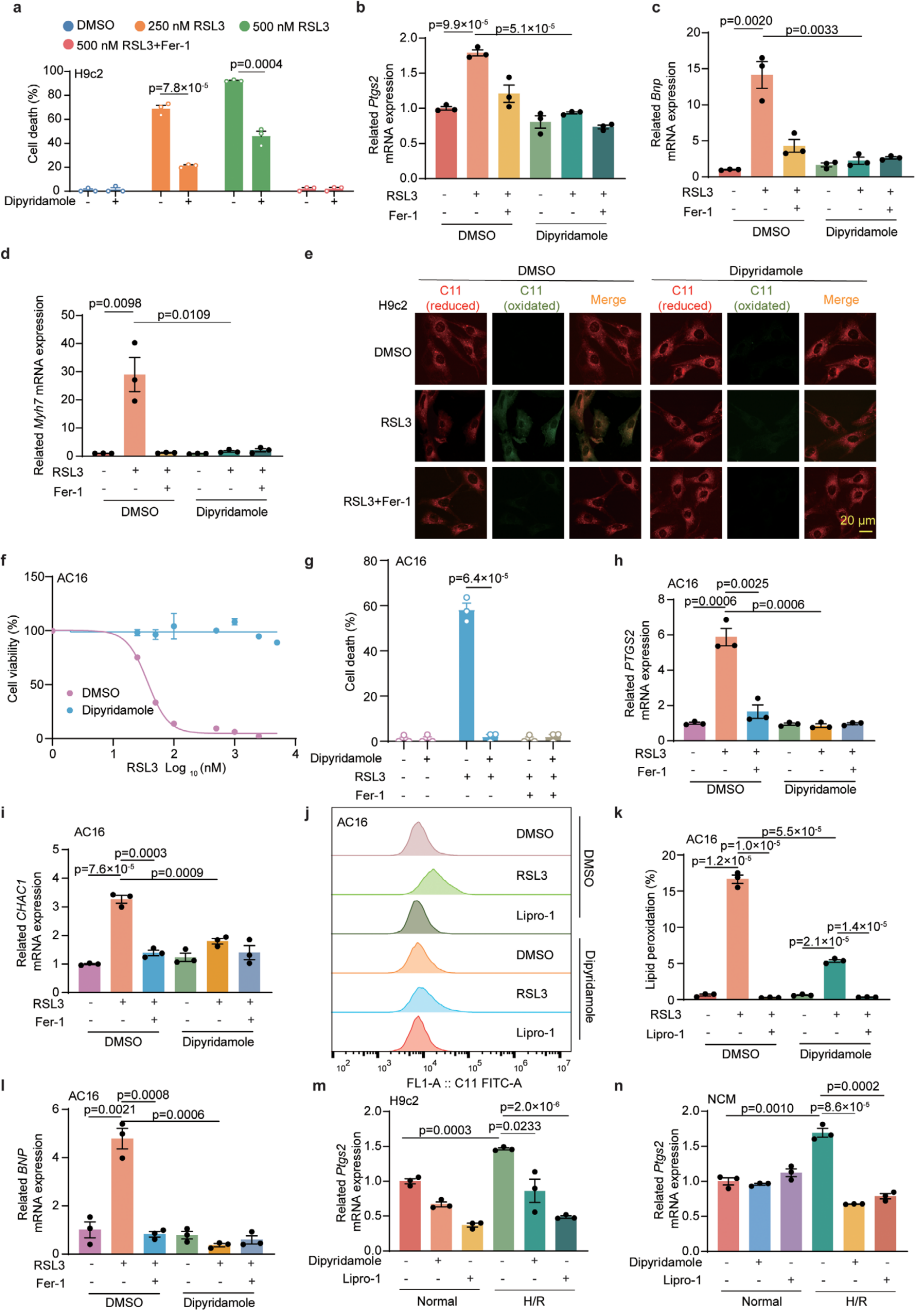
Dipyridamole inhibits ferroptotic cell death in myocardium cells. a) Cell death measurement of H9c2 cells treated with RSL3 (250  and 500 nm), dipyridamole (10 µm), or Fer‐1 (4 µm) for 4 h. Dead cells were labeled with SYTOX Green. b–d) The relative mRNA levels of *Ptgs2* (b), *Bnp* (c), and *Myh7* (d) were quantified by qRT‐PCR in H9c2 cells. e) Immunofluorescence staining of BODIPY 581/591 C11 to detect the levels of lipid peroxidation in the H9c2 cells treated with RSL3 (250 nm), Fer‐1(4 µm) and dipyridamole (10 µm) for 4 h. f) Dose‐dependent toxicity of RSL3‐induced cell death in AC16 cells after pre‐treatment with dipyridamole (10 µm) for 12 h. Cell viability was assessed 12 h post‐treatment after using CCK8. g) Cell death measurement of AC16 cells treated with RSL3 (200 nm), dipyridamole (10 µm), and Fer‐1 (4 µm) for 6 h. Dead cells were labeled with SYTOX Green. h,i) The relative mRNA levels of *PTGS2* (h) and *CHAC1* (i) were quantified by qRT‐PCR in AC16 cells treated with DMSO, RSL3 (200 nm), dipyridamole (10 µm), and Fer‐1 (4 µm) for 3 h. j,k) BODIPYTM 581/591 C11 staining of lipid peroxidation in AC16 cells treated with DMSO, RSL3 (200 nm), Lipro‐1(4 µm) or dipyridamole (10 µm) for 3 h. l) The relative mRNA level of *BNP* was quantified by qRT‐PCR in AC16 cells treated with DMSO, RSL3 (400 nm), dipyridamole (10 µm), or Fer‐1 (4 µm) for 3 h. m,n) The relative mRNA level of *Ptgs2* was quantified by qRT‐PCR in H9c2 cells (m) and NCM (n) pretreated with Lipro‐1 (4 µm) or dipyridamole (10 µm) for 4 h, followed by hypoxia/reoxygenation (H/R). Data and error bars are mean ± SEM, n  =  3 biologically independent experiments in a–d and f‐n. All *P* values were calculated using a two‐tailed, unpaired Student's *t*‐test.

As dipyridamole has been utilized for the treatment of ischemic cardiomyopathy, we investigated whether dipyridamole protects cardiomyocytes from ferroptosis. Therefore, we employed the rat cardiomyocyte cell line H9c2 and neonatal rat cardiomyocytes (NCM) for in vitro experiments to test the function of dipyridamole. Consistent with the aforementioned findings, dipyridamole was found to markedly protect H9c2 and NCM from ferroptosis (**Figure** **2**a; Figure S2a,b, Supporting information).

Furthermore, we assessed the relative mRNA expression of prostaglandin‐endoperoxide synthase 2 (*Ptgs2*), which is a classical marker of ferroptosis. The data showed that supplementation with dipyridamole significantly decreased the level of *Ptgs2* (Figure 2b; Figure S2c, Supporting information). Alongside common indicators of myocardial damage like brain natriuretic peptide (BNP), and myosin heavy chain 7 (MYH7), we observed that dipyridamole, as well as Fer‐1 significantly alleviated myocardial damage (Figure 2c,d; Figure S2d, Supporting information). Consistent results of protein levels of BNP, MYH7, and Creatine kinase myocardial band (CK‐MB) were observed (Figure S2e–h, Supporting information). Moreover, we utilized the BODIPY 581/591 C11 fluorescent probe to determine intracellular levels of lipid peroxidation, which was measured by confocal microscopy (Figure 2e; Figure S2i, Supporting information). Notably, in H9c2 cells, Fer‐1 was effective in shielding against RSL3‐induced lipid peroxidation. Similar results were observed in human cardiomyocytes (Figure 2f–l). Thus, it can be concluded that dipyridamole potently protects cardiomyocytes from ferroptosis‐induced myocardial damage.

Hypoxia goes along with the occurrence of ischemic cardiomyopathy and drives lipid peroxidation and cell death of cardiomyocytes.^[^ ^31^, ^32^ ^]^ In line with these studies, we found that hypoxia treatment in both H9c2 cells and NCM elevated the level of lipid peroxidation and the expression of *Ptgs2* (Figure 2m,n; Figure S2j, Supporting information), whereas dipyridamole robustly reversed these phenotypes. In short, our data uncovered that dipyridamole is effective in protecting myocardial cells against various conditions‐induced ferroptosis.

### 2.3 Dipyridamole‐Mediated Ferroptosis Depends on SLC7A11

**Figure 3:**
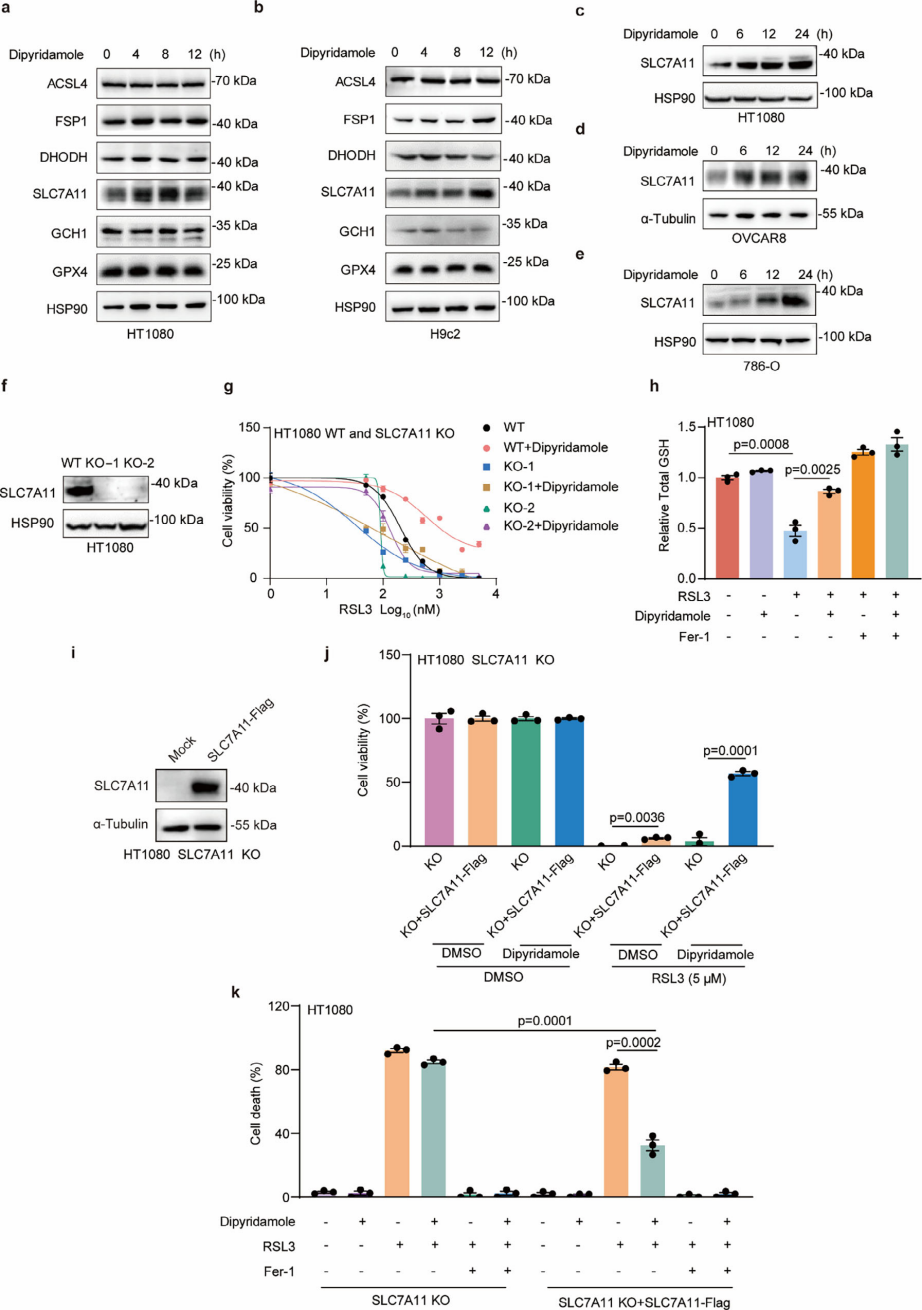
Dipyridamole‐mediated ferroptosis depends on SLC7A11. a,b) Immunoblot assays of classical ferroptosis‐related genes in HT1080 (a) and H9c2 (b) cells with dipyridamole (20 µm) treatment at indicated times. c‐e) Immunoblot assays of SLC7A11 expression in HT1080 (c), OVCAR8 (d), and 786‐O (e) cells with dipyridamole (20 µm) treatment at indicated times. f) Immunoblot assays of SLC7A11 expression in HT1080 WT and SLC7A11 KO cells. g) Cell viability assay in HT1080 WT and SLC7A11 KO cells treated with DMSO, dipyridamole (20 µm), or RSL3 at the indicated concentration. h) The GSH levels in HT1080 cells treated with RSL3 (250 nm), Fer‐1(4 µm), and dipyridamole (10 µm) for 4 h. i) Immunoblot assays of exogenous SLC7A11 expression in HT1080 SLC7A11 KO cells stably expressing with the Mock and SLC7A11‐Flag vector. j) Cell viability assay in HT1080 SLC7A11 KO cells stably expressing with the Mock and SLC7A11‐Flag vector treated with DMSO, RSL3 (5 µm), and dipyridamole (10 µm) at the indicated concentration. k) The cell death of HT1080 SLC7A11 KO cells stably expressing the Mock and SLC7A11‐Flag vector was measured. These cells were treated with DMSO, RSL3 (5 µm), or dipyridamole (10 µm) for 6 h. Dead cells were labeled with SYTOX™ Green. Data and error bars are mean ± SEM, n  =  3 biologically independent experiments in g, h, j, and k. All *P* values were calculated using a two‐tailed, unpaired Student's t‐test.

To explore the molecular mechanism of dipyridamole‐mediated ferroptosis inhibition, we first investigated whether the classical ferroptosis pathways are involved in the role of dipyridamole. We analyzed genes in HT1080 and H9c2 cells associated with ferroptosis‐related processes, including ACSL4, FSP1, DHODH, SLC7A11, GCH1, and GPX4 (**Figure** **3**a,b). Our findings indicated that SLC7A11 exhibited the most significant increase among these ferroptosis‐related genes. We further confirmed the elevated protein expression of SLC7A11 in HT1080, OVCAR8, and 786‐O cells after dipyridamole treatment (Figure 3c–e). Notably, the expression of SLC7A11 was increased in a time‐dependent manner.

To elucidate the impact of SLC7A11 on dipyridamole‐mediated ferroptosis suppression, we utilized CRISPR‐Cas9 technology to generate SLC7A11 KO cell lines (Figure 3f). Interestingly, the protective effect of dipyridamole was largely abolished in SLC7A11 KO cells (Figure 3g). Moreover, we observed a significant decrease of total glutathione (GSH) in cells upon RSL3 treatment, which could be rescued by dipyridamole and Fer‐1 (Figure 3h). As GPX4 utilizes GSH to diminish oxidized lipids, we speculated whether dipyridamole loses its function to defend against ferroptosis in GPX4 KO cells. As expected, dipyridamole was unable to inhibit ferroptotic cell death in GPX4 KO cells (Figure S3a,b, Supporting information). In contrast, deficiency of FSP1, GCH1, or DHODH exhibited little effect on the function of dipyridamole (Figure S3c–h, Supporting information). This suggested that dipyridamole acts as the activator of SLC7A11‐mediated GSH biosynthesis pathway to prevent ferroptotic cell death. Moreover, we found that whereas the protective effect of dipyridamole was largely abolished in HT1080 SLC7A11 KO cells, re‐expressing SLC7A11 in HT1080 SLC7A11 KO cells rescued the phenotype of dipyridamole (Figure 3i–k).

The transcription factor NRF2 plays a critical role in transcriptional upregulation of *SLC7A11*.^[^ ^33^, ^34^, ^35^ ^]^ However, RNA‐Seq and qRT‐PCR analysis showed that the mRNA level of *SLC7A11* was unaffected upon dipyridamole treatment (Figure S3i,j, Supporting information), indicating that dipyridamole could protect cells from death by post‐transcriptionally upregulating SLC7A11.Dipyridamole was used to prevent blood clots after heart valve replacement surgery. Dipyridamole gained antiplatelet function via inhibiting cAMP targeted‐phosphodiesterase such as phosphodiesterase 3 (PDE3) and phosphodiesterase 4 (PDE4).^[^ ^36^ ^]^ Inactivation of phosphodiesterase inhibits adenosine uptake and promotes intracellular cAMP levels.^[^ ^37^ ^]^ We therefore examined whether the role of dipyridamole in combating ferroptosis is related to these processes. As shown in Figure S4a–d (Supporting information), supplementation with cAMP or adenosine was unable to alter the ferroptotic sensitivity or influence the effect of dipyridamole. Roflumilast is a selective PDE4 inhibitor and cilostazol is a PDE3 inhibitor, both of which inhibit adenosine uptake. Notably, neither of these drugs affected the efficacy of dipyridamole in defending against ferroptosis (Figure S4e–h, Supporting information). In addition, dipyridamole was also reported to inhibit the uptake of inosine, a downstream product of adenosine.^[^ ^38^ ^]^ However, we observed that supplementation with exogenous inosine was incapable of regulating the protective effect of dipyridamole against ferroptosis (Figure S4i,j, Supporting information). Taken together, it appears that the protective effect of dipyridamole is dependent on SLC7A11, independent of its previously reported mechanism.

### 2.4 Dipyridamole Stabilizes SLC7A11 via Regulation of RNF126

**Figure 4:**
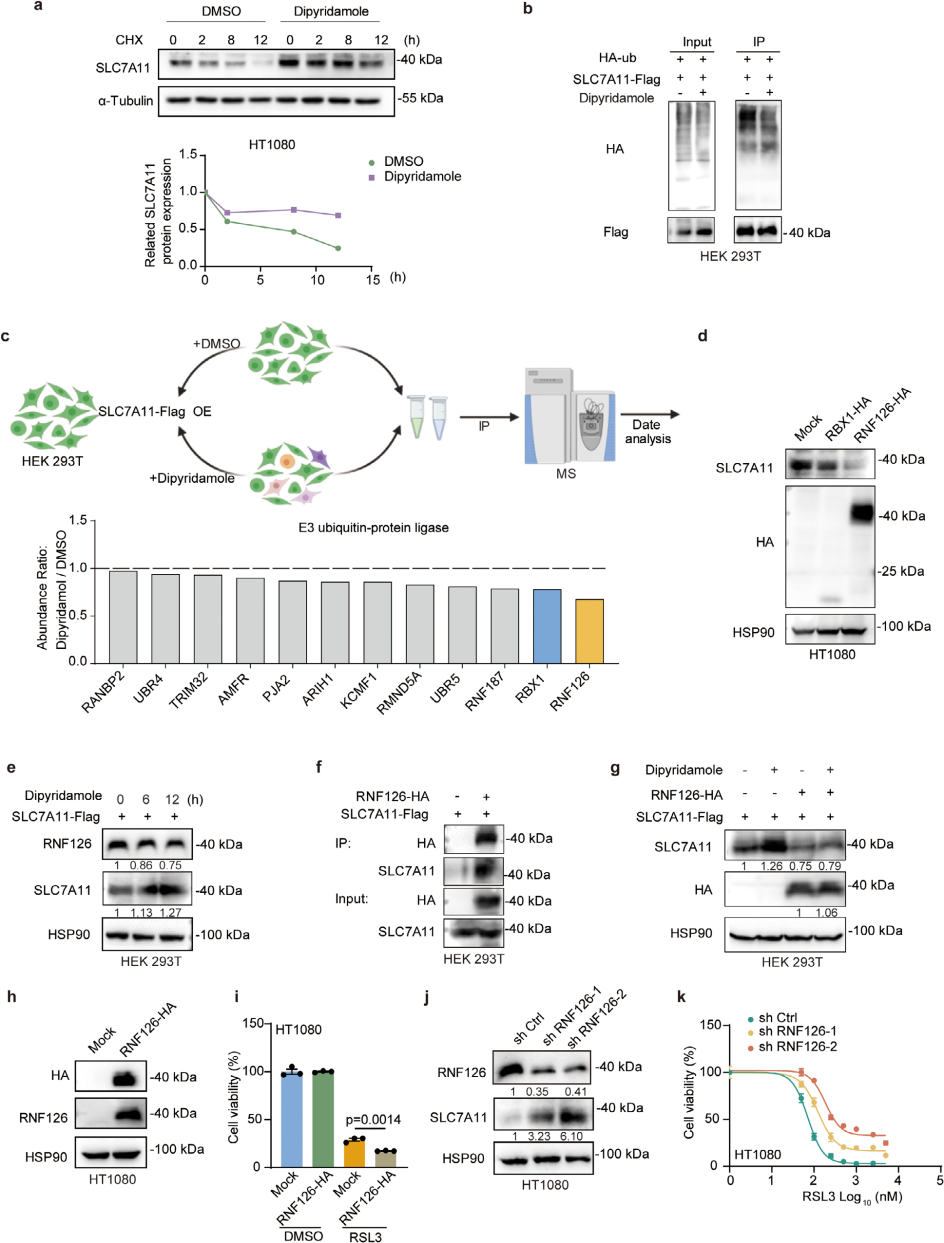
Dipyridamole stabilizes SLC7A11 via regulation of RNF126. a) Immunoblot assays were conducted for SLC7A11 in HT1080 cells which were treated with dipyridamole (20 µm) and CHX (20 mg mL^−1^) for the indicated times (shown in the **up**). Then, the densitometry quantification of SLC7A11 protein levels was calculated by using ImageJ software (NIH), and the results were plotted in the **bottom** panel for the determination of the half‐life. The statistical results are normalized with the data at 0 h in each group. b) Immunoprecipitation assays of exogenous SLC7A11‐Flag and HA‐ubiquitin in HEK 293T cells. c) Pattern diagram of mass spectrometry in HEK 293T cells transfected with SLC7A11‐Flag with or without dipyridamole (10 µm) treatment. Anti FLAG M2 affinity gel was used for immunoprecipitation. d) Immunoblot assays of endogenous SLC7A11 in HT1080 cells transfected with the Mock, RBX1‐HA or RNF126‐HA vector. e) Immunoblot assays of endogenous RNF126 and exogenous SLC7A11 in HEK 293T cells with dipyridamole (20 µm) treatment at 0, 6, and 12 h. f) Immunoblot assays of co‐immunoprecipitation of exogenous RNF126‐HA and SLC7A11‐Flag in HEK 293T cells. g) Immunoblot assays of exogenous RNF126 and SLC7A11 in HEK 293T cells with dipyridamole (20 µm) treatment for 16 h. h) Immunoblot assays of exogenous RNF126‐HA in HT1080 cells transfected with the Mock and RNF126‐HA vector. i) RSL3 (500 nm)‐induced cell death of HT1080 transfected with the Mock and RNF126‐HA vector. Cell viability was assessed 12 h post‐treatment after using CCK8. j) Immunoblot assays of endogenous RNF126‐HA in HT1080 cells infected with the sh Ctrl and sh RNF126 virus. k) Dose‐dependent toxicity of RSL3‐induced cell death of HT1080 sh Ctrl and sh RNF126 cells for 12 h. Cell viability was assessed 12 h post‐treatment after using CCK8. Data and error bars are mean ± SEM, n  =  3 biologically independent experiments in i and k. *P* values were calculated using a two‐tailed, unpaired Student's t‐test. Grayscale analysis of the images was performed using ImageJ software.

The aforementioned data implied that dipyridamole upregulates SLC7A11 in a post‐transcriptional manner. To gain further insight into the mechanism by which dipyridamole upregulates SLC7A11, we first treated cells with protein synthesis inhibitor cycloheximide (CHX) to examine the half‐life of SLC7A11. Whereas CHX induced a rapid decrease of SLC7A11 protein levels in HT1080 cells, treatment of dipyridamole remarkably extended the half‐life of SLC7A11 (**Figure** **4**a). Similar data was achieved in OVCAR8 cells (Figure S5a, Supporting information). We then investigated whether dipyridamole upregulates SLC7A11 by affecting the proteasome degradation pathway. We found that both exogenous and endogenous ubiquitination levels were significantly reduced upon dipyridamole treatment (Figure 4b; Figure S5b,c, Supporting information). These results demonstrated that dipyridamole could inhibit the ubiquitination of SLC7A11. To further investigate the mechanism of how dipyridamole mediates the ubiquitination of SLC7A11, we performed immunoprecipitation (IP) experiments and subsequent protein mass spectrometry analysis (Figure 4c). Interestingly, we identified that the abundance of E3 ligase proteins RBX1 and RNF126 decreased upon dipyridamole treatment. To elucidate whether these E3 ligase proteins contribute to dipyridamole‐mediated SLC7A11 regulation, we first examined the change of SLC7A11 upon ectopic expression of these genes. As shown in Figure 4d, RNF126 exhibited better efficiency to downregulate SLC7A11. Furthermore, consistent with the data from protein mass spectrometry, dipyridamole could significantly reduce the protein expression level of RNF126 (Figure 4e; Figure S5d, Supporting Information). Further Co‐IP experiments revealed that RNF126 can bind with SLC7A11 (Figure 4f). Moreover, overexpression of RNF126 can eliminate upregulation of SLC7A11 protein induced by dipyridamole and also increase susceptibility to ferroptotic cell death (Figure 4h,i). RNF126‐mediated SLC7A11 downregulation was abolished by proteasome inhibitor MG132 (Figure S5e, Supporting information). To further verify the functionality of RNF126, we knocked‐down RNF126 in HT1080 cells and found that the loss of RNF126 significantly upregulated SLC7A11 expression and inhibited ferroptosis (Figure 4j,k). In contrast, re‐expressing RNF126 in these RNF126 knockdown (KD) cells reversed these phenotypes (Figure S5e,f, Supporting information). Additionally, Co‐IP experiments demonstrated that overexpression of RNF126 in wild type (WT) cells elevated the ubiquitination levels of SLC7A11 and reduced its protein levels. In contrast, the loss of RNF126 decreased the ubiquitination levels of SLC7A11 and upregulated its expression. Notably, re‐expression of RNF126 in RNF126 KD cells restored these phenotypes (Figure S5g, Supporting information). Overall, our results uncovered that dipyridamole‐mediated upregulation of SLC7A11 is at least partially through a decrease of RNF126.

### 2.5 Dipyridamole Prevents Against I/R‐Induced and Dox‐Induced Cardiac Damage

**Figure 5:**
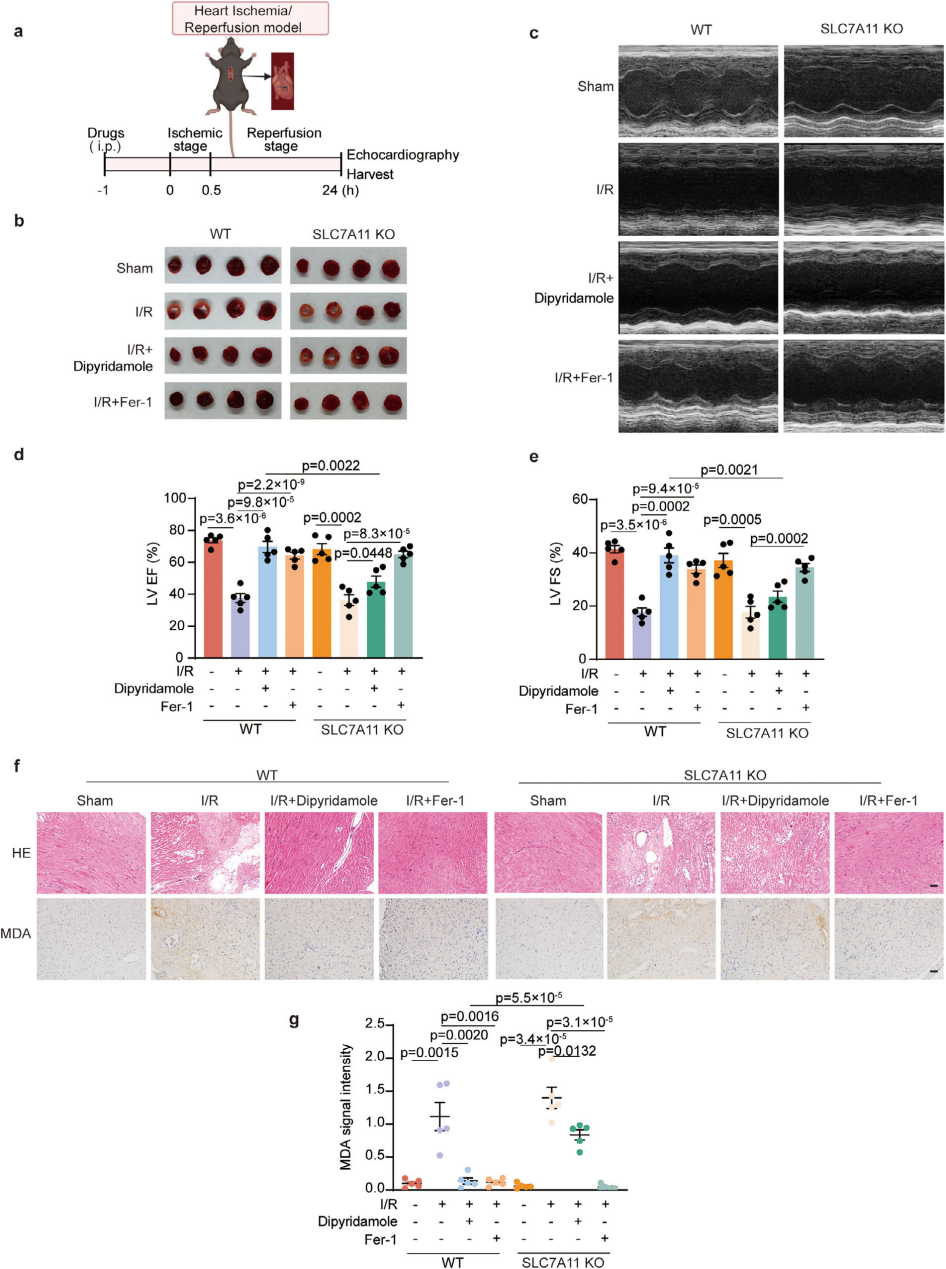
Dipyridamole prevents I/R‐induced cardiac damage. a) Pattern diagram of myocardial ischemia‐reperfusion in mice. WT and SLC7A11 KO*C57BL/6J* mice were intraperitoneally injected with vehicle, Fer‐1(10 mg kg^−1^), or dipyridamole (10 mg kg^−1^) for 1 h, followed by 30 min of ischemia and 24 h of reperfusion. b) Representative photographs of TTC staining in WT and SLC7A11 KO mice heart sections in the indicated groups are from (a). (n = 5 mice per group). c) Representative images of WT and SLC7A11 KO mice echocardiography in the indicated groups are from (a). (n = 5 mice per group). d,e) Left ventricular ejection fraction (LVEF) (d) and left ventricular fractional shortening (LVFS) (e) of WT and SLC7A11 KO mice in the indicated groups are from (a). (n = 5 mice per group). f) Representative images of heart HE staining and IHC staining of MDA in the indicated groups are from (a). Scale bars, 50 µm. g) MDA intensity was scored in the indicated groups from (f). (×100, n = 5 mice per group). Data and error bars are mean ± SEM, n  =  5 biologically independent experiments in d, e, and g. All *P* values were calculated using a two‐tailed, unpaired Student's *t*‐test.

Overall, we have substantiated the role and mechanism of dipyridamole in vitro. We intended to further corroborate the role of dipyridamole in vivo. As dipyridamole has been used for ischemic heart disease, we therefore first performed experiments on I/R‐induced cardiac injury (**Figure** **5**a). Within the myocardial I/R model, we successfully visualized the 2,3,5‐triphenyltetrazolium chloride (TTC) staining indicating the inflicted damage (Figure 5b). As expected, dipyridamole displayed a strong protective effect as well as Fer‐1 in WT mice. However, unlike Fer‐1, the protective effect of dipyridamole was greatly diminished in SLC7A11 KO mice. We then conducted the assessment of mice's myocardial function using echocardiography, with left ventricular ejection fraction (LVEF) and left ventricular fractional shortening (LVFS) standing as indices of myocardial damage (Figure 5c–e). Consistent with the data of TTC staining, both Fer‐1 and dipyridamole enabled to maintain of cardiac function (Figure 5c–e). On the contrary, only Fer‐1, not dipyridamole, rescued the symptoms in SLC7A11 KO mice post I/R. Furthermore, hematoxylin‐eosin (HE) staining illustrated that cardiac function was impaired in both WT and SLC7A11 KO mice after I/R; however, dipyridamole was able to rescue myocardial function insult in WT mice, but not in KO mice (Figure 5f). We further validated the role of dipyridamole via the immunohistochemical (IHC) staining of malondialdehyde (MDA), which is a byproduct of lipid peroxidation to serve as a marker of ferroptosis. The IHC results exhibited that Fer‐1 managed to block lipid peroxidation and ferroptosis inflicted by I/R in both WT and KO mice (Figure 5f,g). On the contrary, dipyridamole could solely provide protection against myocardial ischemia‐reperfusion injury (IRI) in WT mice, but not in SLC7A11 KO mice. In conclusion, our data revealed that dipyridamole acts as a potent ferroptosis inhibitor to prevent myocardial IRI via SLC7A11.

Accumulating evidence indicates that the clinical anti‐cancer drug Dox has been found to induce cardiac damage and even death via promoting ferroptosis.^[^ ^23^ ^]^ Therefore, the question arises as to whether dipyridamole mitigates Dox‐induced myocardial injury. As shown in Figure S6a (Supporting information), we used the model of Dox‐induced cardiotoxicity. Various assessments including echocardiography of LVEF and LVFS (Figure S6b–d, Supporting information), the mRNA levels of *Bnp*, *Myh7* (Figure S6e,f, Supporting information), the upregulation of *Ptgs2* and MDA staining (Figure S6g–i, Supporting information), indicated that dipyridamole and Fer‐1 could safeguard the WT mice myocardium against Dox‐induced ferroptosis and injury. As shown in Kaplan‐Meier survival curves of mice (Figure S6j, Supporting information), Dox administration resulted in severe death of mice, whereas both Fer‐1 and dipyridamole significantly increased the survival rate. Further experiments elucidated that dipyridamole, not Fer‐1, lost the ability to rescue the SLC7A11 KO mice from Dox‐induced death (Figure S6k, Supporting information). Together, these findings uncovered that dipyridamole efficiently alleviates I/R‐ or Dox‐induced cardiac injury via SLC7A11‐mediated ferroptosis inhibition.

### 2.6 A General Role for Dipyridamole‐Mediated Protection in Various Tissues

**Figure 6:**
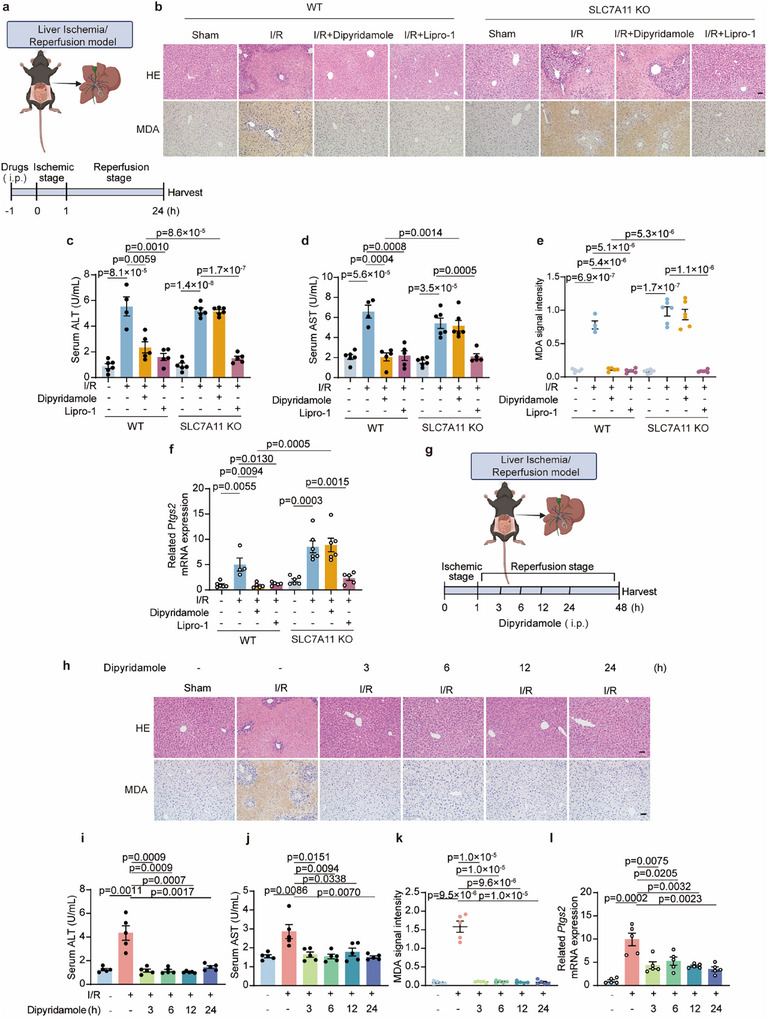
Dipyridamole prevents I/R‐induced liver damage. a) Pattern diagram of liver ischemia‐reperfusion in mice. WT and SLC7A11 KO mice were intraperitoneally injected with vehicle, Lipro‐1(10mg kg^−1^), or dipyridamole (10 mg kg^−1^) for 1 h, subjected to 1 h of ischemia, then followed by reperfusion for 24 h. b) Representative images of liver HE staining and IHC staining of MDA in the indicated groups are from (a). Scale bars, 50 µm. (×100). Figure represents a total of n  =  6 (WT+sham+vehicle), 4 (WT+I/R +vehicle), 5 (WT+I/R + dipyridamole), 5 (WT+I/R + Lipro‐1), 6 (SLC7A11 KO+sham+vehicle), 6 (SLC7A11 KO+I/R +vehicle), 6 (SLC7A11 KO+I/R + dipyridamole), and 5 (SLC7A11 KO+I/R + Lipro‐1) mice per group. c,d) Serum ALT and AST were measured in the indicated groups from (a). The number of mice per group in this figure is consistent with Figure (b) above. e) MDA intensity was scored in the indicated groups from (b). (×100). The number of mice per group in this figure is consistent with Figure (b) above. f) The relative mRNA level of *Ptgs2* was quantified by qRT‐PCR in the indicated groups from (a). The number of mice per group in this figure is consistent with Figure (b) above. g) Pattern diagram of liver ischemia‐reperfusion in mice. WT mice were subjected to 1 h of ischemia, and then intraperitoneally injected with dipyridamole (10 mg kg^−1^) at indicated times, then reperfusion for 48 h. h) Representative images of liver HE staining and IHC staining of MDA in the indicated groups are from (g). Scale bars, 50 µm. (n = 5 mice per group). i,j) Serum ALT and AST were measured in the indicated groups from (g).(n = 5 mice per group). k) MDA intensity was scored in the indicated groups from (h). (×100, n = 5 mice per group). l) The relative mRNA level of *Ptgs2* was quantified by qRT‐PCR in the indicated groups from (g). (n = 5 mice per group). Data and error bars are mean ± SEM, n  =  6 (WT+sham+vehicle), 4 (WT+I/R +vehicle), 5 (WT+I/R + dipyridamole), 5 (WT+I/R + Lipro‐1), 6 (SLC7A11 KO+sham+vehicle), 6 (SLC7A11 KO+I/R +vehicle), 6 (SLC7A11 KO+I/R + dipyridamole), and 5 (SLC7A11 KO+I/R + Lipro‐1) mice in c–f ; n  =  5 mice in i–l. All *P* values were calculated using a two‐tailed, unpaired Student's *t*‐test.

To further validate whether the role of dipyridamole is a common phenomenon in organ injury, we used the I/R model to examine the effect of dipyridamole in the liver and kidney. In the liver I/R model, we observed liver damage as well as a significant increase in serum alanine aminotransferase (ALT) and aspartate aminotransferase (AST) levels, which acts as the index of liver injury (**Figure** **6**a–d). In the meantime, both dipyridamole and Lipro‐1 could markedly protect the liver from I/R‐induced injury (Figure 6b–d). Analysis of the change of ferroptosis markers MDA and *Ptgs2* indicated dipyridamole strongly inhibited lipid peroxidation and blocked ferroptosis (Figure 6b,e,f). Consistent with our aforementioned data, the protective effect of dipyridamole was substantially blocked in SLC7A11 KO mice (Figure 6b–f). We also performed kidney I/R experiments and found that supplementation with dipyridamole significantly alleviated kidney damage indicated by HE staining, blood urea nitrogen (BUN), and serum Creatinine (Cre) levels (Figure S7a–d, Supporting information), which are the markers of kidney injury. Additionally, administration of dipyridamole could efficiently decrease ferroptosis indicated by MDA, *Ptgs2*, and glutathione‐specific gamma‐glutamylcyclotransferase 1(*Chac1)* (Figure S7b,e–g, Supporting information).

In terms of clinical significance, we aimed to investigate whether dipyridamole can also be used to prevent organ damage after I/R insult. Following different durations (3, 6, 12, and 24 h) after liver I/R, we administered dipyridamole to the mice (Figure 6g). Interestingly, the HE staining of the mouse liver demonstrated that dipyridamole could efficiently rescue liver injury after insult (Figure 6h). Furthermore, the increase of serum ALT and AST after insult was significantly blocked by the administration of dipyridamole (Figure 6i,j). Moreover, histological MDA staining and qRT‐PCR results provided evidence that dipyridamole alleviated ferroptosis in the liver after I/R (Figure 6k,l). In addition, we also explored the role of dipyridamole in the Dox‐induced myocardial and liver injury model after insult. In accordance with the aforementioned data, dipyridamole exhibited a viable effect in defending against Dox‐induced cardiac and liver damage after insult (Figure S8a–k, Supporting information). In conclusion, our results suggested that dipyridamole plays a general role in protecting tissues from I/R or Dox‐induced injury before or after insult.

## 3 Discussion

Tissue homeostasis is essential to maintain normal organism function and the physiological balance of the body. Disrupting tissue homeostasis via metabolism disorder, drug‐ or I/R‐induction leads to severe inflammation, organ dysfunction, and tissue failure.^[^ ^39^, ^40^ ^]^ Fundamentally, these symptoms are due in large part to excess cell death. Inhibiting cell death has been thought of as an effective approach to tissue injury treatment. Accumulating evidence reveals that ferroptosis contributes to various types of tissue injury and suppressing ferroptosis via ferroptosis inhibitors could achieve an efficient therapeutic effect to attenuate tissue damage,^[^ ^1^, ^41^ ^]^ implying that targeting ferroptosis is a potential therapeutic strategy for tissue injury. However, there is an unavoidable issue that no clinical ferroptosis inhibitor has been applied in disease treatment. Whether clinical compounds could act as potent ferroptosis suppressors has been poorly understood.

Here, we identify that dipyridamole significantly abolishes lipid peroxidation and ferroptotic cell death. Interestingly, the clinical trial corroborated the therapeutic potential of dipyridamole in many diseases, such as NCT00457405‐Ischemia‐Reperfusion Injury, NCT03688815‐ Ischemic Heart Disease, NCT00760708‐ Ischemia and Coronary Disease, NCT00430170‐ Ischemia‐Reperfusion Injury, and NCT00767663‐Coronary Heart Disease. Moreover, the clinical trial corroborates the therapeutic potential of dipyridamole in acute respiratory distress syndrome patients, demonstrating improved outcomes with dipyridamole adjunctive therapy.^[^ ^42^ ^]^ It should be noted that I/R induced tissue injury and Coronary Heart Disease are closely related to ferroptosis,^[^ ^16^, ^22^, ^23^, ^25^, ^43^, ^44^, ^45^ ^]^ as supplementation with ferroptosis inhibitors significantly suppresses tissue damage. Indeed, our study showed that dipyridamole displays a good therapeutic effect for I/R‐ and drug‐induced tissue injury in the preclinical mouse model as well as Fer‐1 or Lipro‐1. Most previous studies reveal that pre‐administration of ferroptosis inhibitors before inducing injury model efficiently prevents tissue injury.^[^ ^17^, ^46^ ^]^ However, whether these compounds are also effective after insult is little investigated. It should be noted that tissue injury has occurred in a large part of patients before taking precautions. Our findings confirm that dipyridamole significantly attenuates tissue injury after insult. From a clinical perspective, our findings indicate that tr eating tissue injury by therapeutic administration of dipyridamole may efficiently help prevent disease development before or after insult.

GPX4‐GSH pathway occupies the dominant position in suppressing ferroptosis among multiple defense systems.^[^ ^9^ ^]^ GPX4 utilizes reduced glutathione to obliterate oxidized lipids, thereby inhibiting ferroptosis both in vitro an in vivo.^[^ ^17^, ^47^ ^]^ The major substrate for glutathione biosynthesis is cysteine, which is strictly regulated by cystine transporter SLC7A11. Genetic ablation or pharmacological inhibition of SLC7A11 via erastin, imidazole ketone erastin, sulfasalazine, or sorafenib rapidly decreases the level of GSH and confers to ferroptosis.^[^ ^1^, ^48^, ^49^, ^50^ ^]^ Whereas sorafenib acts as a clinical drug to efficiently inhibit liver cancer development via inactivation of SLC7A11, the clinical compound identified to prevent tissue injury via activation of SLC7A11 is little known. Our work showed that dipyridamole renders cells resistant to ferroptosis and alleviates tissue injury via activation of SLC7A11. Deficiency of SLC7A11 severely weakens the protective effect of dipyridamole both in vitro and in vivo, suggesting that dipyridamole is a potent SLC7A11 activator to defend against ferroptosis.

## 4 Experimental Section

The study on mice complied with all relevant ethical regulations for animal experiments. All experimental protocols were approved by the Institutional Animal Care and Use Committee of Shandong University.

### Chemicals and Reagents

The Immunology/Inflammation Compound Library (L4100) was purchased from Selleck. Ferroptosis‐related chemicals: RSL3 (TargetMol, T3646); Ferrostatin‐1(Selleck, S7243); Liproxstatin‐1 (Selleck, S7699); 3‐Methyladenine (TargetMol, T1879); Z‐VAD(OMe)‐FMK (TargetMol, T6013); Necrostatin‐1 (Selleck, S8034); Erastin (Selleck, S7242); ML210 (TargetMol, T8375); ML162 (Selleck, S4452). Other Chemicals: Dipyridamole (Selleck, S1895); Mometasone furoate (Selleck, S1987); Cyclic AMP (MCE, HY‐B1511); Adenosine (Selleck, S1647); Adenosine (MCE, HY‐B0228); Roflumilast (MCE, HY‐15455); Cilostazol (MCE, HY‐17464); Inosine (MCE, HY‐N0092); Doxorubicin (Adriamycin) HCl (Selleck, S1208); Cycloheximide (Selleck, S7418); MG132 (Selleck, S2619).

### Cell Lines and Cell Culture

HT1080, A375, 786‐O, HEK 293T, AC16, and H9c2 cell lines were purchased from the ATCC. All cells were grown in DMEM (Thermo Fisher) supplemented with 10% fetal bovine serum (Biological Industries, Israel), 1% penicillin, and 1% streptomycin (Biological Industries, Israel) at 37 °C with 5% CO_2_ and have been proven to be negative for mycoplasma contamination. HT1080 GPX4 KO cells need Fer‐1 (2 µm) supplementation daily to culture; HT1080 SLC7A11 KO cells need β‐mercaptoethanol (Sigma, 21985023, 55 µm) supplementation daily to culture. HT1080 DHODH KD cells need Uridine (TargetMol, T2221, 50 µm) supplementation daily to culture.

### Isolation and Culture of NCM

NCM were isolated from 2‐3‐day‐old rats. The hearts were dissected from the rats using ophthalmic forceps and then roughly minced using ophthalmic scissors. Subsequently, the minced heart tissues were further dissected in D‐hanks solution and transferred to conical tubes equipped with rotors. Collagenase II (Sigma, C6885) at a concentration of 0.75 mg mL^−1^ was added, and the tubes were placed on a magnetic stirrer until tissue dissolution occurred. The suspended cells were then centrifuged and resuspended in a cell culture medium. The cells were plated in 10 cm^2^ dishes, allowing fibroblasts to adhere. 2 h later, the upper layer of cells, mainly consisting of cardiomyocytes, was collected. Cardiomyocytes were seeded at a density of 2 × 10^5^ cells per well in 24‐well plates and cultured for an additional 48 h before further experimentation. The myocardial cell culture medium was composed of 81% DMEM (Macgene, CM10014), 10% fetal bovine serum, 6% horse serum, 1% BRDU (Sigma, B5002), 1% penicillin, and 1% streptomycin.

### Plasmid Construction and Transfection

For the plasmids, sh Ctrl, sh RNF126, sh RBX1, SLC7A11‐Flag, RBX1‐HA, RNF126‐HA, pCMV‐Flag vector, pCMV‐Flag vector, and pCMV‐HA vector were purchased from MIAOLING BIOLOGY. Full‐length SLC7A11 was cloned into a Plenti‐Flag vector.

For the sgRNAs, these sgRNAs were constructed using lentiCRISPR‐v2 (Addgene, 52961). The following primers are listed in the following Table.

**Table:** 

Gene	Forward primer [5′‐3′]	Reverse primer [5′‐3′]
Human‐DHODH‐sg1	CACCGGTGACTCC AAAACCTCAGGA	AAACTCCTGAGG TTTTGGAGTCACC
Human‐FSP1‐sg1	CACCGCACTCTC ATTCACTCCCAAG	AAACCTTGGGAG TGAATGAGAGTGC
Human‐GCH1‐sg1	CACCGAACCAAG TGATGCTCACACA	AAACTGTGTGAGC ATCACTTGGTTC
Human‐SLC7A11‐sg1	CACCGAAGGGCG TGCTCCAGAACAC	AAACGTGTTCTG GAGCACGCCCTTC
Human‐SLC7A11‐sg2	CACCGCTTGCAT ATGTATATCCATG	AAACCATGGATA TACATATGCAAGC

### Preparation of Lentivirus particles

A lentiviral packaging system consisting of lentiviral plasmid, psPAX2 (Addgene,12260), and pMD2.G (Addgene, 12259) was co‐lipofected into 293T cells in a 3:2:1 molar ratio using LentiFit (HANBIO, HB‐LLF‐1000) at a final volume of 200 µL per well according to the manufacturer's instructions. The cell culture supernatants were pooled, and centrifuged at 10, 000 g for 5 min. The supernatant was carefully collected and then filtered through a 0.45 µm syringe filter. The filtered supernatant was stored at −80 °C until further use.

### Cell Viability Assay

The cells were seeded in the 96‐well plates and allowed to reach 60% confluency. After ≈12 h, the cells were treated with different concentrations of the compounds and RSL3 for the indicated times. The Cell Counting Kit‐8 (CCK‐8) (TargetMol, C0005) was then added to the wells and incubated for 30 min. The absorbance at 450 nm was measured to determine cell viability.

### Cell Death Assay

HT1080, 786‐O, A375, OVCAR8, H9c2, AC16, and NCM cells were seeded in 24‐well plates and subjected to drug pretreatment. After 12 h, the cells were treated with RSL3 for the indicated times. SYTOX Green (Invitrogen, S7020) was then added at a dilution of 1:30000 (avoiding light exposure) at 37 °C for 15 min. Fluorescence imaging was taken to visualize the cells, using a magnification of 20×, and three replicate wells were randomly counted.

### Flow Cytometry

HT1080 or AC16 cells were seeded on 24 well plates and allowed to adhere for 12 h. The cells were pre‐treated with DMSO, dipyridamole (10 µm), or Lipro‐1 (4 µm) for 12 h. Subsequently, the cells were subjected to RSL3 exposure for 3 h. Following treatment, the cells were harvested and washed twice with phosphate‐buffered saline (PBS). BODIPY 581/591 C11 (5 µm) (Thermo Fisher Scientific, D3861) was then applied and incubated at 37 °C for 30 min. After incubation, the cells were washed twice with PBS. Finally, lipid ROS levels were analyzed by a Becton Dickinson FACS Calibur machine through the FL1 channel. The data were analyzed by FlowJo version 10 software. A minimum of 5000 cells were analyzed in each sample.

### Immunofluorescence of BODIPY 581/591 C11 Staining

H9c2 or NCM cells were seeded on 24 well plates and allowed to adhere for 12 h. The cells were pre‐treated with DMSO, dipyridamole (10 µm), or Fer‐1(4 µm) for 12 h. Subsequently, the cells were subjected to RSL3 exposure for 4 h or 1% O_2_ for 2 h, reoxygenation for 2 h. Following treatment, the cells were washed twice with phosphate‐buffered saline (PBS). BODIPY 581/591 C11 (5 µm) (Thermo Fisher Scientific, D3861) was then applied and incubated at 37 °C for 30 min. After incubation, the cells were washed twice with PBS and fixed with 4% paraformaldehyde for 10 min. Finally, the cells were imaged using a high‐speed confocal platform (40×) (Andor, Dragonfly 200). (Oxidized states were indicated by green fluorescence, while reduced states were observed in red.)

### Immunoblot Assays

After obtaining the samples, the cells and tissues were treated with radioimmunoprecipitation assay (Beyotime, P0013D) lysate buffer supplemented with protease and phosphatase inhibitors (APE×BIO, K1007) in a 1:100 ratio. The human primary antibodies detailed antibody information as follows: α‐Tubulin (1: 2000; Proteintech, 11224‐1‐AP), β‐actin (1: 2000; ZSGB‐BIO, TA‐09), ACSL4 (1: 1000; Abcam, ab155282), DHODH (1: 1000; Proteintech, 14877‐1‐AP), FLAG (1: 1000;Cell Signaling Technology,14793), FLAG (1: 1000;Invitrogen, PA1‐984B), FSP1 (1: 1000; Proteintech, 20886‐1‐AP), GCH1 (1: 1000; Proteintech, 28501‐1‐AP), GCLM (1: 1000; Abcam, ab126704), GPX4 (1: 1000; Abcam, ab125066), HA (1: 1000; Cell Signaling Technology, 3724), HMOX1 (1: 1000; Cell Signaling Technology, 70081), HSP90 (1: 2000; ZSGB‐BIO, TA‐12), MYH7 (1: 2000; Proteintech, 22280‐1‐AP), NQO1 (1: 1000; Proteintech, 11451‐1‐AP), PTGS2 (1: 1000; Proteintech, 27308‐1‐AP), NRF2 (1: 1000; Abcam, ab137550), RNF126 (1: 1000; Proteintech, 66647‐1‐Ig), SLC7A11 (1: 1000; Cell Signaling Technology, 12691), SLC7A11 (1: 1000; Abcam, ab307601), Ubiquitin (1: 1000; Cell Signaling Technology, 3936),Ubiquitin (1: 1000; Santa Cruz Biotechnology, sc‐166553). The NC membrane (Pall, 66485) was incubated overnight at 4 °C. Horseradish peroxidase‐labeled secondary antibodies from Jackson ImmunoResearch Laboratories (1: 5000; 111‐035‐003 and 115‐035‐003) were then added and incubated with the membranes for 1 h at room temperature. Finally, the protein bands were visualized using an ECL kit from Tonon (180‐506) and imaged on a gel imager with serial exposure.

### RNA‐Seq Analysis

HT1080 cells were seeded on 10 cm^2^ plates and allowed to adhere for 12 h. Then the cells were treated with DMSO or dipyridamole (10 µm) for 6 h. Following treatment, the cells were washed twice with phosphate‐buffered saline (PBS). Finally, 1 × 10^6^ cells per group were collected and frozen in liquid nitrogen. RNA‐Seq was analyzed by Beijing Novogene Biotechnology Co., Ltd.

### Quantitative PCR (qRT‐PCR) Analysis

The individual mRNA levels were normalized to β‐actin or GAPDH mRNA levels for each well. The data were then analyzed using the 2−ΔΔCT method, with the average of the control group. The TRNzol Universal Total RNA Extraction Reagent (TIANGEN, DP424) was used to extract RNA from tissues or cell samples. 1000 ng of RNA was reverse transcribed (Vazyme, R223‐01). The resulting cDNA was then used for qRT‐PCR analysis with the ChamQ SYBR Color Qpcr Master Mix (Vazyme,027E3270BB). The qRT‐PCR primers are listed in the following Table. The primers were synthesized by Sangon Biotech (Shanghai) Co., Ltd.

**Table:** 

Mouse	Forward	Reverse
β‐actin	CAACTTGATGTATGAAGGCTTTGGT	ACTTTTATTGGTCTCAAGTCAGTGTACAG
Bnp	AAGTCCTAGCCAGTCTCCAGA	GAGCTGTCTCTGGGCCATTTC
Chac1	GGTCATTGCCACACAGATCC	GTGCTCATCTTGTGCCTGAG
Gapdh	GGAGCGAGATCCCTCCAAAAT	GGCTGTTGTCATACTTCTCATGG
Myh7	GCTGAAAGCAGAAAGAGATTATC	TGGAGTTCTTCTCTTCTGGAG
Ptgs2	GGGAGTCTGGAACATTGTGAA	GTGCACATTGTAAGTAGGTGGACT
Rat	Forward	Reverse
Gapdh	GCATCTTCTTGTGCAGTGCC	TACGGCCAAATCCGTTCACA
Bnp	AAGGACCAAGGCCCTACAAA	AACAACCTCGCCCGTCAC
Myh7	ATGCGGAAGTGGTAGCTGC	CCGGTGATGAGGATGGACTG
Ptgs2	CTCAGCCATGCAGCAAATCC	GGGTGGGCTTCAGCAGTAAT
Human	Forward	Reverse
GAPDH	GGAGCGAGATCCCTCCAAAAT	GGCTGTTGTCATACTTCTCATGG
SLC7A11	TCTCCAAAGGAGGTTACCTGC	AGACTCCCCTCAGTAAAGTGAC
PTGS2	CTGGCGCTCAGCCATACAG	CGCACTTATACTGGTCAAATCCC
CHAC1	GAACCCTGGTTACCTGGGC	CGCAGCAAGTATTCAAGGTTGT
BNP	GTCTGGCCGGACACTCAG	TGCACTGGTGTCTTCAACAAC

### CK‐MB and BNP Levels Measurement

H9c2 cells were exposed to RSL3, Fer‐1 (4 µm) or dipyridamole (10 µm) for 6 h. 1 ×  10^6^ cells per sample were collected and intracellular CK‐MB or BNP levels were determined using the Rat CK‐MB (Creatine Kinase MB Isoenzyme) ELISA Kit (Elabscience, E‐EL‐R1327) and Rat BNP (Brain Natriuretic Peptide) ELISA Kit (E‐EL‐R0126) according to the manufacturer's instructions.

### GSH Levels Measurement

HT1080 cells were exposed to RSL3 (250 nm), Fer‐1 (4 µm), or dipyridamole (10 µm) for 8 h. 3 ×  10^5^ cells per sample were collected, and intracellular GSH levels were determined using the GSSG/GSH Quantification Kit II (DOJINDO, G263) according to the manufacturer's instructions.

### Liquid Chromatography‐Mass Spectrometry/Mass Spectrometry (LC‐MS/MS)

After transfecting SLC7A11‐Flag into HEK 293T cells for 36 h, the cell lysate was prepared as follows: 20 mL of 1 m Tris‐HCl (pH = 7.3), 20 mL of 5 m NaCl, 200 mL of 50% glycerol, 0.4 mL of 0.5 m EDTA and DDW to 1 L. Anti‐FLAG M2 affinity gel (Sigma, A2220) was then added to the lysate and incubated at 4 °C with gentle rotation for 8 h to enrich SLC7A11‐Flag onto the beads. Following this, wash five times to ensure purification prior to commencing mass spectrometry analysis.

In the cell lysate of HT1080 cells, ctrl and biotin‐dipyridamole probes (20 µm) were incubated for 4 h at 4 °C. The Pierce Streptomycin avidin agarose (Sigma, 20353) was added and allowed to incubate for 8 h at 4 °C with gentle rotation. Following this, wash five times to ensure purification prior to commencing mass spectrometry analysis.

Resuspend the beads in 400 µL of 25 mm ammonium bicarbonate, then wash them 5 times. Resuspend the pelleted beads in 200 µL of ammonium bicarbonate containing 10 mm dithiothreitol for 40 min at 55 °C with rotation at 1000 rpm. After reaching room temperature, add 40 mm iodoacetamide to the suspension and rotate at 1000 rpm in the dark for 30 min at room temperature. Add 250 ng of sequencing‐grade trypsin to the supernatant, leaving the reaction overnight (16 h) at 37 °C with rotation at 1000 rpm in the dark. Pierce c18 spin columns were used for peptide desalting. Add 200 µl of 50% acetonitrile (ACN) to rinse the walls of the spin column and wet the resin. Centrifuge at 1500 g for 1 min. Discard flow‐through and repeat once to fully activate the C18 resin bed. Add 200 µL of 0.5% trifluoroacetic acid (TFA) in 5% ACN, and centrifuge at 1500 g for 1 min. Discard flow‐through and repeat once. Add 2% TFA in 20% ACN 1 µL for every 3 µL of the sample. Load the sample on top of the resin bed, place the column into a receiver tube, and centrifuge at 1500 g for 1 min. To ensure complete binding, recover flow‐through and repeat steps twice. Add 200 µL of 0.5% TFA in 5% ACN to the column and centrifuge at 1500 g for 1 min. Discard flow‐through and repeat three times. Place the column in a new receiver tube. Add 20 µl of 70% ACN to the top of the resin bed. Centrifuge at 1500 g for 1 min and repeat three times. Dry the combined sample in an evaporator. Re‐dissolve the dried sample in 20 µl of ultrapure water plus 0.1% formic acid. Measure the peptide concentration using a nanodrop at 205 nm. Adjust the sample concentration to 250 ng µL^−1^. Centrifuge at 14000 g for 5 min and transfer supernatant to sample vial.LC‐MS/MS analysis was performed on an Easy nLC 1200 (Thermo Fisher Scientific, Bremen, Germany) coupled to an Orbitrap Fusion Lumos equipped with a nanospray flex ion source (Thermo Fisher Scientific, Bremen, Germany). The MS raw data for each sample were combined and searched using the Proteome Discoverer 2.5 software (Thermo Fisher Scientific, Bremen, Germany) for identification and quantitation analysis.

### Proteomic LC‐MS/MS Analysis

LC‐MS/MS analysis was performed on an Easy nLC 1200 (Thermo Fisher Scientific, Bremen, Germany) coupled to an Orbitrap Fusion Lumos equipped with a nanospray flex ion source (Thermo Fisher Scientific, Bremen, Germany). Mobile phase A contains 0.1% formic acid (v/v) in water; mobile phase B contains 0.1% formic acid in 80% ACN.

The peptides were dissolved in water with 0.1% formic acid and separated on a commercial RP–HPLC pre–column (75 µm × 2 cm) (Thermo, 164535) and an RP–HPLC analytical column (75 µm × 25 cm) (Thermo, 164941), both packed with 2 µm C18 beads. A linear gradient was used, ranging from 10–30% B in 90 min, then a linear increase from 30–50% B in 10 min, and finally a linear increase to 100% B in 10 min, at a flow rate of 300 nL min^−1^.

The Orbitrap Fusion Lumos acquired data in a data‐dependent manner alternating between full‐scan MS and MS2 scans. The spray voltage was set at 2.1 kV and the temperature of ion transfer capillary was 320 °C. The MS1 spectra (350–2000 m/z) were collected with 60 000 resolution (at m/z 200), AGC target of 1 × 10^6^, and 50 ms maximum injection time. Selected ions were sequentially fragmented in a 3 s cycle by HCD with 0% normalized collision energy, quadrupole isolation windows of 1.6 m/z, at 15000 resolution (at m/z 200). AGC target of 1 × 10^5^ and 22 ms maximum injection time were used. Dynamic exclusion was set to 45 s and only precursors with charge states 2+ to 7+ were isolated for MS/MS experiments.

The MS raw data for each sample were combined and searched using the Proteome Discoverer 2.5 software (Thermo Fisher Scientific, Bremen, Germany) for identification and quantitation analysis. Abundance ratios were calculated using the pairwise ratio approach. The peptide group ratios were calculated by taking the geometric median of all combinations of the ratios from all the replicates regarding the selected study factor, specifically the treatment. Subsequently, the protein ratios were calculated as the geometric median of the peptide group ratios.

### Animals

The SLC7A11 KO mice were gifts from Dr. Fudi Wang at Zhejiang University School of Medicine. The SLC7A11 KO mice and their WT littermates (8‐12 weeks) on a *C57BL/6J* background were used in study. *C57BL/6J* mice were purchased from Vital River Laboratory Animal Technology (Beijing, China).

### Cardiac IRI Model

8–12 weeks old WT or SLC7A11 KO male mice were randomly divided into different groups. For mice in the model group, after anesthesia, the hearts were exposed between the 3rd and 4th ribs of the left sternum, and the left anterior descending (LAD) coronary artery was ligated with a 6–0 silk ligature. After occlusion for 30 min, the coronary artery was reperfused by removing the knot of the suture for 24 h. Mice in the sham group received the same procedure except for LAD ligation. After 24 h of IRI, echocardiography was performed and the mice were then euthanized to harvest heart and blood samples for further experiments.

### Partial Liver IRI Model

8–12 weeks old WT or SLC7A11 KO male mice were used in the model of hepatic I/R. Briefly, the mice were first treated with anesthesia, the arterial and portal venous blood supply to the median and left lateral lobes were interrupted by an atraumatic clip. After 1 h of local ischemia, the clip was removed. Mice were sacrificed after 24 h of reperfusion. The sham‐operated mice underwent the same procedure, but without interrupting the atraumatic clip. In the treatment groups, mice were infused at 1 h prior to the onset of liver ischemia with the vehicle, dipyridamole (10 mg kg^−1^, i.p.) or Lipro‐1 (10 mg kg^−1^, i.p.).

In the study for the therapeutic effect of dipyridamole, mice were treated with dipyridamole (10 mg kg^−1^, i.p.) at 3, 6, 12, and 24 h after I/R. After 48 h of IRI, the mice were euthanized to harvest liver and blood samples for further experiments.

### Kidney IRI Model

8‐week‐old WT male mice were first treated with anesthesia and both kidney pedicles were identified through two small paramedial dorsal incisions and clamped for 30 min. I/R was confirmed by observing the color change of the kidney. They were placed on a heating pad to maintain core body temperature at 37 °C after surgery. The sham‐operated mice underwent the same surgical procedure without kidney pedicles clamped. For the treatment group, intraperitoneal injections of dipyridamole (10 mg kg^−1^, i.p.) or vehicle given 1 h prior to the onset of kidney ischemia. After 24 h, blood and kidney tissues were collected for further experiments.

### Dox‐Induced Acute Liver and Cardiac Injury Mice Model

In the Dox‐induced cardiotoxicity model, 8‐week‐old WT and SLC7A11 KO male mice were pretreated with vehicle, Fer‐1 (2 mg kg^−1^, i.p.) and dipyridamole (5 mg kg^−1^, i.p.), followed by intraperitoneal injection with vehicle and Dox (20 mg kg^−1^, i.p.) on day 0, then injected with Fer‐1 or dipyridamole once a day. After 4 days of treatment, echocardiography was performed, and the mice were then euthanized to harvest heart and blood samples for further analysis.

In the Dox‐induced acute liver and cardiac injury model, 8‐week‐old WT male mice received a single injection of Dox (20 mg kg^−1^). Then mice were given dipyridamole (10 mg kg^−1^) or vehicle after 3 and 12 h once a day. After 4 days of injection, the mice were euthanized to collect blood, heart, and liver samples for further analysis.

### Echocardiography

Cardiac function in mice was assessed using a small animal color Doppler ultrasound instrument (Vevo2100, VisualSonics, Canada). M‐mode echocardiography was used for visual observation, and LVEF and LVFS were calculated for statistical analysis. At least 3 mice were included in each group for analysis.

### TTC Staining

The mice were euthanized after I/R surgery for 24 h. The hearts were removed and sliced evenly at 1 mm, then stained with 2% TTC (Solarbio, G3005) at 37 °C for 30 min. The normal area was stained red, but the infarcted area was unstained.

### HE and IHC

After fixing fresh tissues in 4% paraformaldehyde for 48 h, perform paraffin embedding. The tissue sections were cut at 4 µm for HE or IHC analysis. Sections were soaked in xylene for dewaxing and graded alcohol for rehydrating and incubated with 2% Tween‐20 for 30 min. The sections were then incubated with the primary antibody for MDA (1:200; Adipogen, JAI‐MMD‐030N) overnight at 4 °C. The mouse/rabbit polymer detection system (ZSGB‐BIO, PV‐9000) and subsequent DAB staining kit (ZSGB‐BIO, ZLI‐9018) were used to detect MDA staining. Dehydrate the sections, mount them with a coverslip, and capture images using a microscope. Each section was randomly selected from 3 fields of view at 20× magnification. Statistical analysis was performed using Image J Proplus software.

### Serum AST and ALT Assays

The serum AST and ALT levels were measured according to the kit (BC1565 and BC1555, Solarbio) instructions. The reaction reagent was added to the samples to be measured as required, and a standard curve with the standards in the kit was calculated. The absorbance of each sample was determined with a microplate reader at 505 nm.

### Cre and BUN Assays

Cre and BUN were measured with the Creatinine Assay kit (sarcosine oxidase) (C011‐2‐1, Jiancheng Bio, Nanjing, China) and the Urea Assay kit (C013‐2‐1, Jiancheng Bio, Nanjing, China). The reaction reagent was added to the samples according to the kit manual. The absorbance of each sample was determined with a microplate reader at a wavelength of 546 nm (Cre) and 640 nm (BUN).

### Statistical Analyses

All the data were shown as the mean ±SEM. Analysis method: two‐tailed Student's t‐tests are used to compare the means between two groups (GraphPad Prism 8). Log‐rank (Mantel‐Cox) tests are used to compare the Kaplan‐Meier survival curves of mice.

## Conflict of Interest

The authors declare no conflict of interest.

## Author Contributions

X.Z, S.S, S.L, and Y.J. these authors contributed equally. X.Z., S.S., S.L. and Y.J. performed the experiments with assistance from B.H., Y.Y., L.Y., X.Y., H.W., C.L., D.S., H.Y., D.Z., Q.S., S.Y, C.Y., J.L., Y.L., J.M., B.C., X.Z., S.S. designed the experiments; B.C. supervised the study, established collaborations, allocated funding for this study. B.C., X.Z. and S.S. wrote most of the manuscript with assistance from Y.N., F.W., L.D. and all authors commented on the manuscript.

## Supporting information



Supporting Information

## Data Availability

The data that support the findings of this study are available on request from the corresponding author. The data are not publicly available due to privacy or ethical restrictions.
