# Supplementary material for: Dipyridamole Acts as Clinical Ferroptosis Inhibitor to Prevent from Tissue Injury
Source: Adv Sci (Weinh). 2025 May 14;12(23):2500566. doi: 10.1002/advs.202500566 (PMC12199345; doi:10.1002/advs.202500566)
Supplement: Supplementary file 1 — Supporting Information [file ADVS-12-2500566-s001.docx]

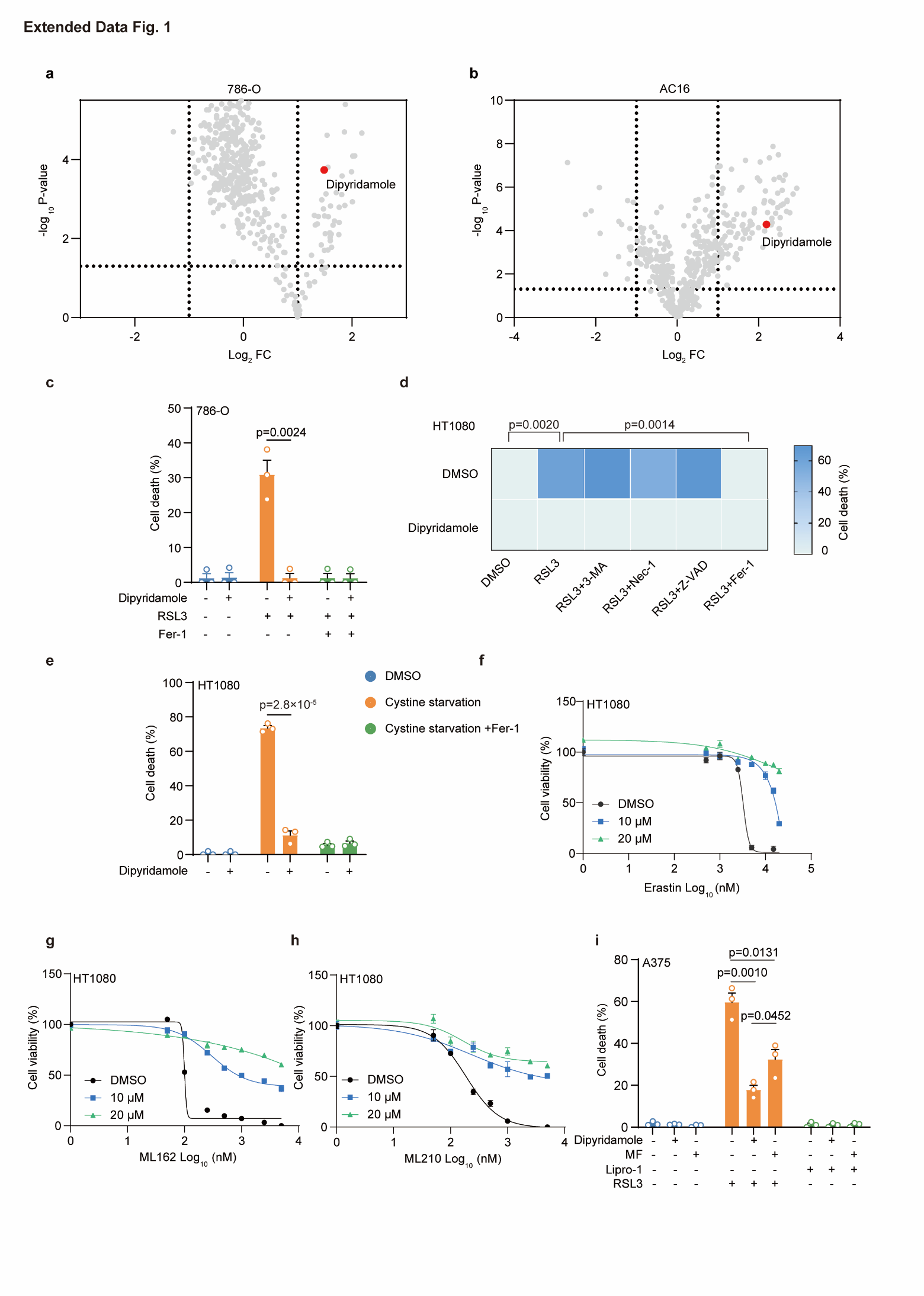


**Extended Data Fig. 1 Dipyridamole is a potent ferroptosis inhibitor in a panel of cell lines.**

**a, b,** Schematic of identification of potential ferroptosis inhibitors for disease treatment, using 786-O (**a**) and AC16 (**b**) cells pretreated with clinical drugs (10 μM) followed by RSL3 (500 nM) treatment for 24 h.

**c,** Cell death measurement of 786-O cells treated with RSL3 (500 nM), dipyridamole (10 μM) or Fer-1 (4 μM) for 4 h. Dead cells were labeled with SYTOX^TM^ Green.

**d,** Heatmap data showing the cell death measurement of HT1080 cells treated with RSL3 (250 nM), 3-MA (2 mM), Nec-1 (4 μM), Z-VAD (20 μM), Fer-1 (4 μM) or dipyridamole (10 μM) for 6 h. Dead cells were labeled with SYTOX^TM^ Green.

**e,** Cell death measurement of HT1080 cells treated with cystine/cysteine starvation, dipyridamole (10 μM) or Fer-1 (4 μM) for 24 h. Dead cells were labeled with SYTOX^TM^ Green.

**f-h,** Cell viability assay in HT1080 cells treated with dipyridamole (10 and 20 μM), erastin (**d**), ML162 (**e**) and ML210 (**f**) for the indicated concentration. Cell viability was assessed after 12 h post-treatment using CCK8.

**i,** Cell death measurement of HT1080 cells treated with RSL3 (500 nM), dipyridamole (10 μM), MF (10 μM) or Lipro-1 (4 μM) for 12 h. Dead cells were labeled with SYTOX^TM^ Green.

Data and error bars are mean ± SEM, n = 3 biologically independent experiments in **a**–**i**. All *P* values were calculated using a two-tailed, unpaired Student’s t-test.


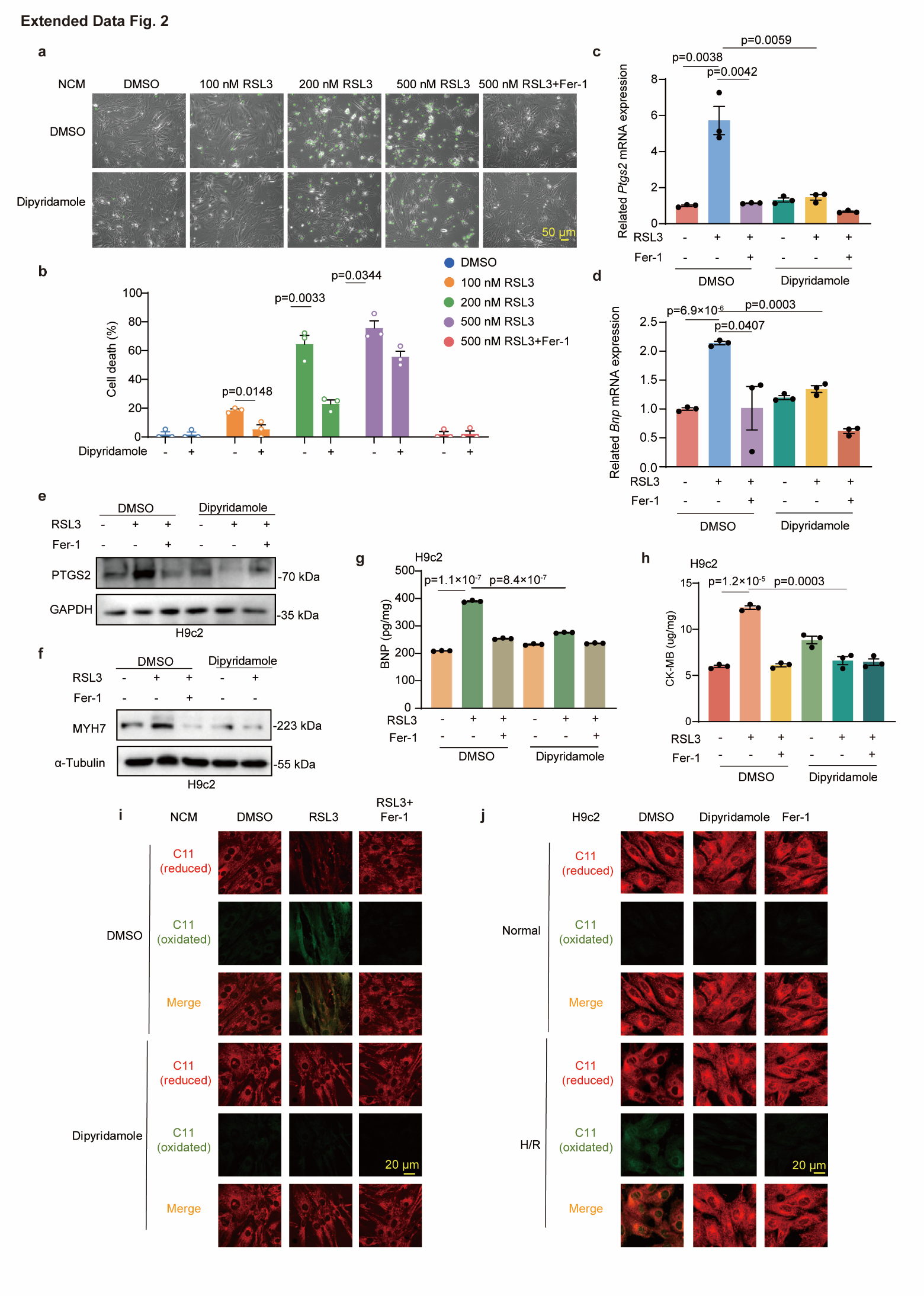


**Extended Data Fig. 2. Dipyridamole inhibits ferroptotic cell death in NCM and H9c2.**

**a,b,** Cell death measurement of NCM treated with DMSO, RSL3 (100 nM, 250 nM and 500 nM), dipyridamole (10 μM) or Fer-1 (4 μM) for 4 h. Dead cells were labeled with SYTOX^TM^ Green.

**c,d,** The relative mRNA levels of *Ptgs2* (**c**) and *Bnp* (**d**) were quantified by qRT-PCR in NCM.

**e,** Immunoblot assays of PTGS2 expression in H9c2 cells with DMSO, dipyridamole (10 μM), RSL3 (150 nM) or Fer-1(4 μM) treatment for 3 h.

**f, I**mmunoblot assays of MYH7 expression in H9c2 cells with DMSO, dipyridamole (10 μM), RSL3 (100 nM) or Fer-1(4 μM) treatment for 3 h.

**g,** ELISA analysis of the levels of BNP treated with RSL3 (400 nM), dipyridamole (10 μM) and Fer-1 (4 μM) for 3 h in H9c2 cells.

**h,** ELISA analysis of the levels of CK-MB treated with RSL3 (100 nM), dipyridamole (10 μM) and Fer-1 (4 μM) for 4 h in H9c2 cells.

**i,** Immunofluorescence staining of BODIPY^TM^ 581/591 C11 to detect the levels of lipid peroxidation in NCM after treatment with DMSO, RSL3 (250 nM), Fer-1(4 μM) or dipyridamole (10 μM) for 4 h.

**j,** Immunofluorescence staining of BODIPY^TM^ 581/591 C11 to detect the levels of lipid peroxidation in H9c2 cells pretreated with DMSO, Fer-1(4 μM) or dipyridamole (10 μM) for 4 h followed by hypoxia/reoxygenation (H/R).

Data and error bars are mean ± SEM, n = 3 biologically independent experiments in **b**–**d**, **g** and **h**. All *P* values were calculated using a two-tailed, unpaired Student’s t-test.


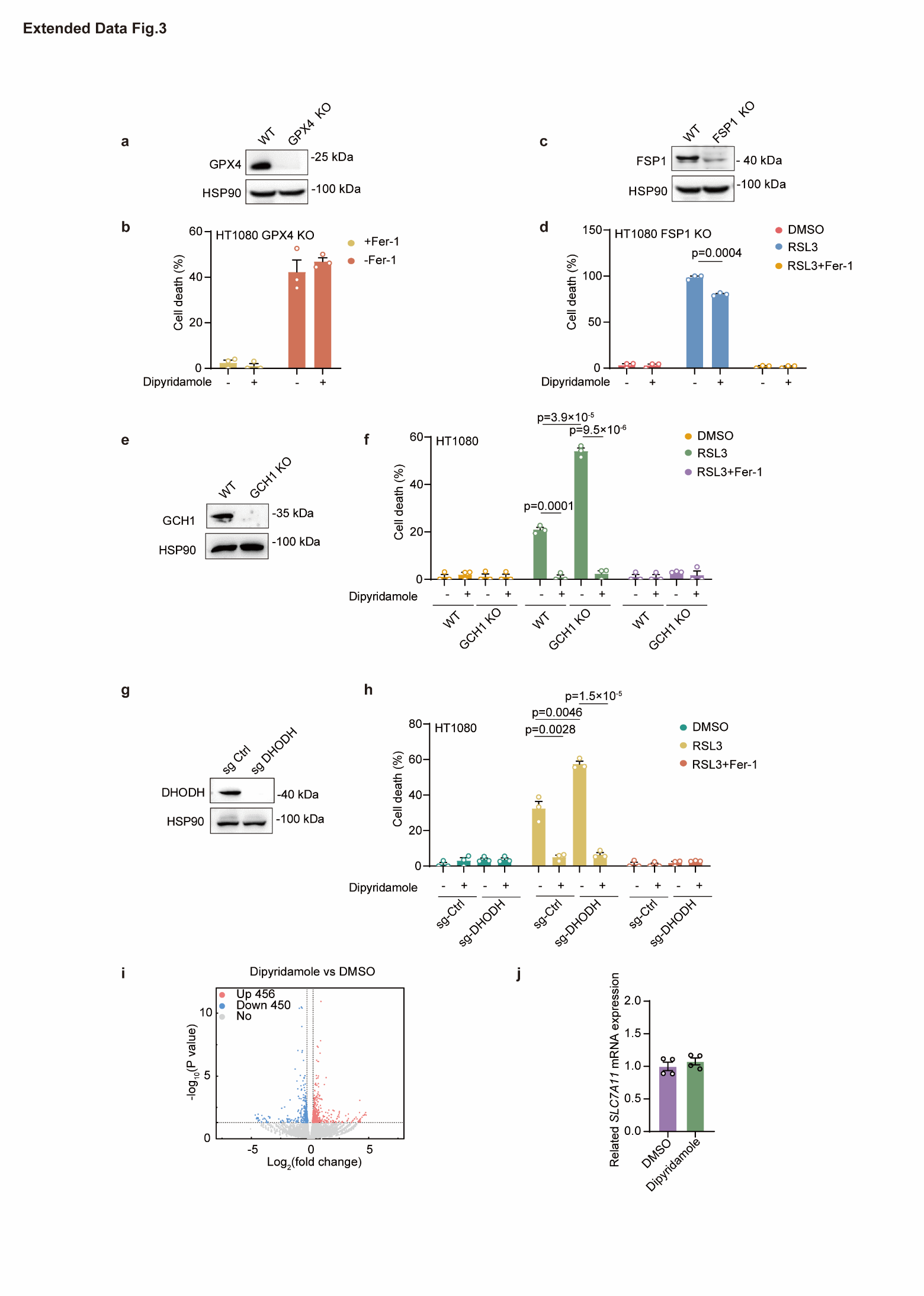


**Extended Data Fig.3. Dipyridamole-mediated ferroptosis inhibition is independent of canonical ferroptosis pathways.**

**a,** Immunoblot assays of GPX4 expression in HT1080 WT and GPX4 KO cells.

**b**, Cell death measurement in HT1080 WT and GPX4 KO cells treated with RSL3 (250 nM), Fer-1 (4 μM) or dipyridamole (10 μM) for 4 h. Dead cells were labeled with SYTOX^TM^ Green.

**c,** Immunoblot assays of FSP1 expression in HT1080 WT and FSP1 KO cells.

**d**, Cell death measurement in HT1080 WT and FSP1 KO cells treated with RSL3 (1 μM), Fer-1 (4 μM) or dipyridamole (10 μM) for 4 h. Dead cells were labeled with SYTOX^TM^ Green.

**e,** Immunoblot assays of GCH1 expression in HT1080 WT and GCH1 KO cells.

**f**, Cell death measurement in HT1080 WT and GCH1 KO cells treated with RSL3 (250  nM), Fer-1 (4 μM) or dipyridamole (10 μM) for 4 h. Dead cells were labeled with SYTOX^TM^ Green.

**g,** Immunoblot assays of DHODH in HT1080 sg Ctrl or sg DHODH cells.

**h,** Cell death measurement in HT1080 sg Ctrl and sg DHODH cells treated with RSL3 (500 nM), Fer-1(4 μM) or dipyridamole (10 μM) for 4 h. Dead cells were labeled with SYTOX^TM^ Green.

**i,** RNA-Seq analysis in HT1080 cells supplemented with DMSO or dipyridamole (10 μM) treatment for 6 h.

**j,** The relative mRNA level of *SLC7A11* was quantified by qRT-PCR in HT1080 cells supplemented with DMSO or dipyridamole (10 μM) for 6 h.

Data and error bars are mean ± SEM, n = 3 biologically independent experiments in **b, d**, **f** and **h**. n = 4 biologically independent experiments in **j.** All *P* values were calculated using a two-tailed, unpaired Student’s t-test.


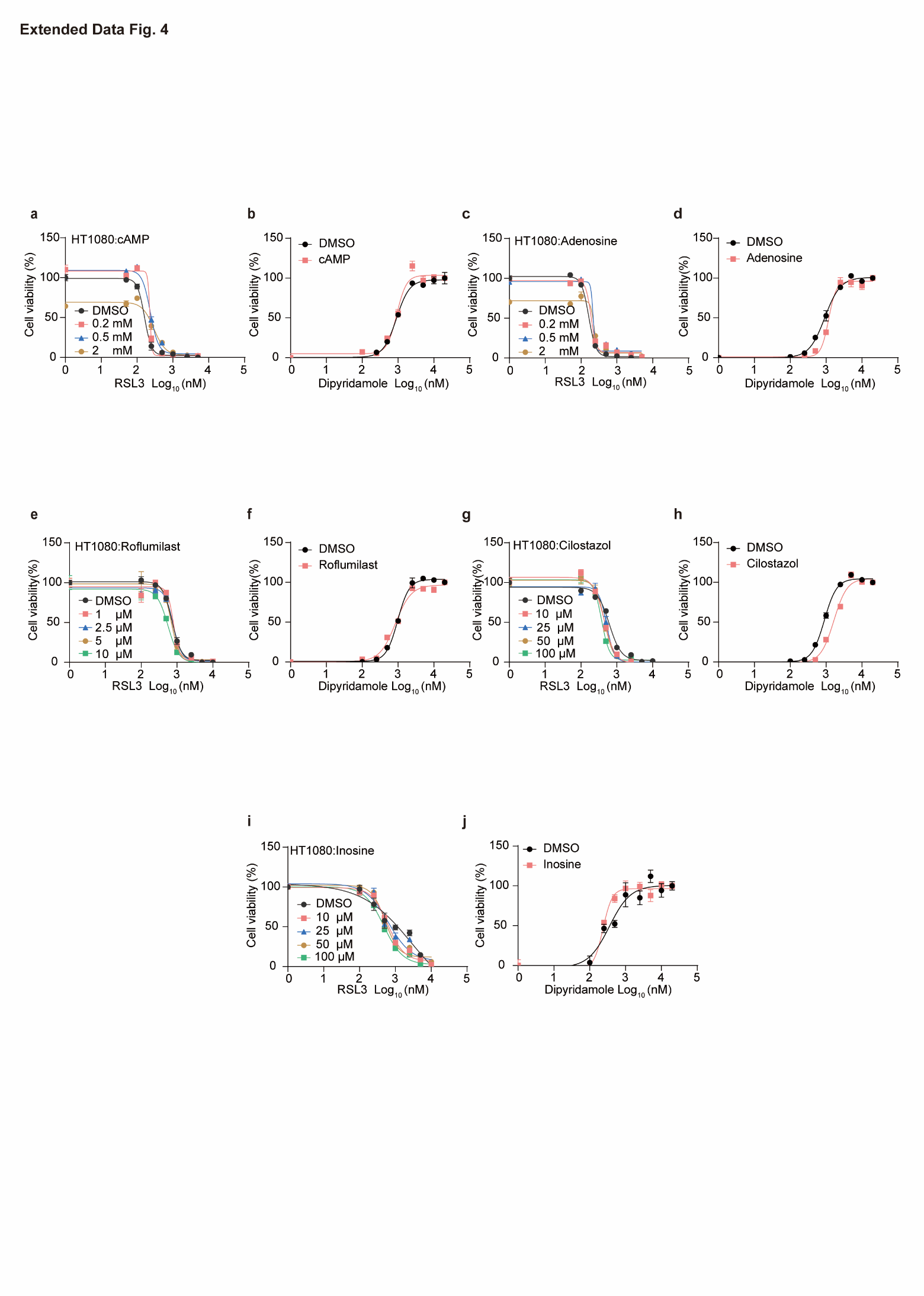


**Extended Data Fig. 4. Dipyridamole-mediated ferroptosis inhibition is independent of cAMP pathways.**

**a,b,** Cell viability assay in HT1080 cells treated with cAMP and RSL3 at the indicated concentration. (**b**) The concentration of cAMP is 0.5 mM.

**c,d,** Cell viability assay in HT1080 cells treated with Adenosine and RSL3 at the indicated concentration. (**d**) The concentration of Adenosine is 0.5 mM.

**e,f,** Cell viability assay in HT1080 cells treated with Roflumilast and RSL3 at the indicated concentration. (**f**) The concentration of Roflumilast is 10 μM.

**g,h,** Cell viability assay in HT1080 cells treated with Cilostazol and RSL3 at the indicated concentration. (**h**) The concentration of Cilostazol is 50 μM.

**i,j** Cell viability assay in HT1080 cells treated with Inosine and RSL3 at the indicated concentration. (**j**) The concentration of Inosine is 50 μM.


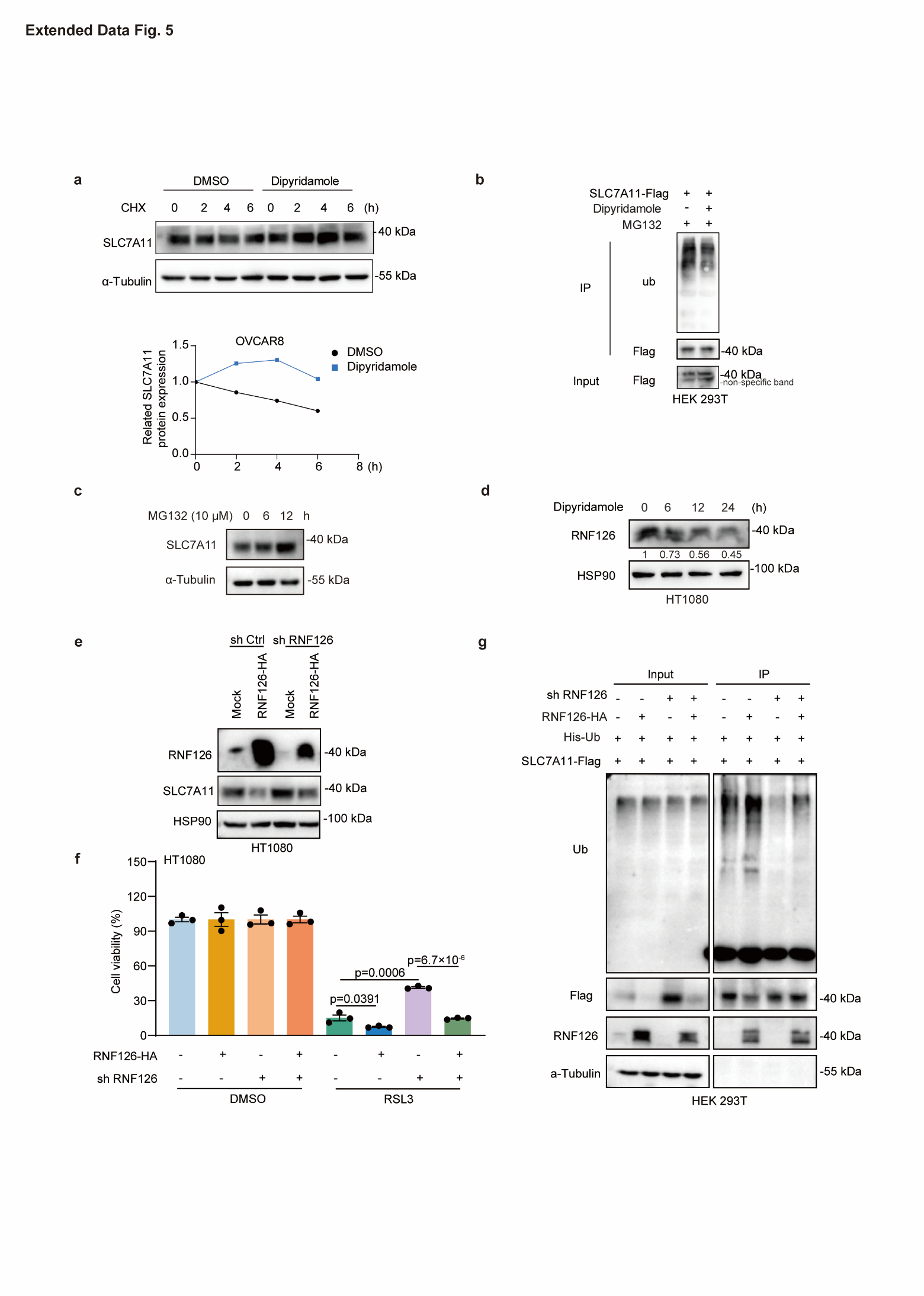


**Extended Data Fig 5. Dipyridamole stabilizes SLC7A11 via regulation of RNF126.**

**a,** Immunoblot assays (up) and quantification (bottom) of SLC7A11 expression in OVCAR8 cells with dipyridamole (20 μM) and CHX (20 ug ml^-1^) treatment at indicated times.

**b,** Immunoprecipitation assays of exogenous SLC7A11-Flag and endogenous ubiquitin in HEK 293T cells treated with MG132 (20 µM) for 6 h.

**c,** Immunoblot assays of SLC7A11 expression in HT1080 cells with MG132 (10 μM) treatment at indicated times.

**d,**Immunoblot assays of endogenous RNF126 in HT1080 cells with dipyridamole (20 μM) treatment at 0, 6,12 and 24 h.

**e,** Immunoblot assays were performed to detect RNF126 expression in HT1080 sh Ctrl and sh RNF126 cells transfected with the Mock and RNF126-HA vector.

**f,** Cell viability assay was performed in HT1080 sh Ctrl and sh RNF126 cells transfected with the Mock and RNF126-HA vector and treated with DMSO, RSL3 (2.5 μM) or dipyridamole (10 μM). Cell viability was measured 12 h post-treatment after using CCK8.

**g,** Immunoprecipitation assays of exogenous SLC7A11-Flag, RNF126-HA and endogenous ubiquitin in HEK 293T cells infected with the sh Ctrl and sh RNF126 virus for 48 h.

Data and error bars are mean ± SEM, n = 3 biologically independent experiments in **f**. All *P* values were calculated using a two-tailed, unpaired Student’s t-test. Grayscale analysis of the images was performed using ImageJ software.


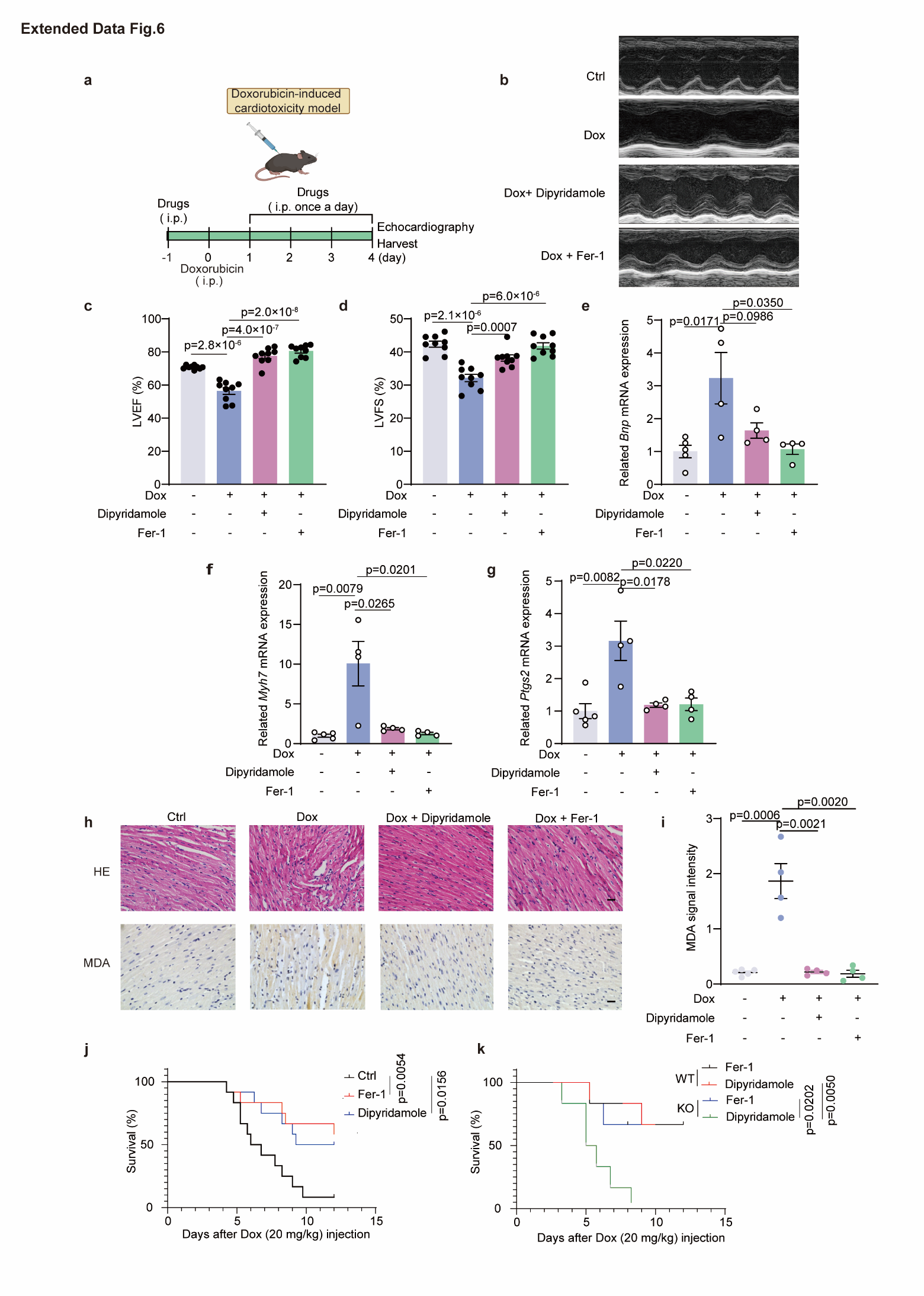


**Extended Data Fig. 6. Dipyridamole prevents against Dox-induced cardiotoxicity.**

**a,** Pattern diagram of Dox-induced cardiotoxicity in mice. WT mice were pretreated with vehicle, Fer-1 (2 mg kg^-1^) or dipyridamole (5 mg kg^-1^), followed by intraperitoneal injection of Dox (20 mg kg^-1^) on day 0, then injected with vehicle, Fer-1 and dipyridamole once a day.

**b,** Representative images of WT mice echocardiography on days 4 in the indicated groups are from (**a**). (n=3 mice per group)

**c,d,** Left ventricular ejection fraction (LVEF) (**c**) and left ventricular fractional shortening (LVFS) (**d**) of WT mice on days 4 in the indicated groups are from (**a**). (n=3 mice per group)

**e-g,** The relative mRNA levels of *Bnp* (**e**), *Myh7* (**f**) and *Ptgs2* (**g**) were quantified by qRT-PCR in mice heart in the indicated groups from (**a**). Figure represents for a total of n=5 (sham+vehicle), 4 (Dox +vehicle), 4 (Dox + dipyridamole) and 4 (Dox+Fer-1) mice per group.

**h,** Representative images of mice heart HE staining and IHC staining of MDA in the indicated groups from (**a**). Scale bars, 50 µm. The number of mice in per group in this figure is consistent with the figure (**e-g**) above.

**i,** MDA intensity was scored in the indicated groups from (**h**). (×100). The number of mice in per group in this figure is consistent with the figure (**e-g**) above.

**j,** Kaplan-Meier survival curves of WT mice pretreated with vehicle, Fer-1 (2 mg kg^-1^) or dipyridamole (5 mg kg^-1^), followed by intraperitoneal injection of Dox (20 mg kg^-1^) on day 0, then injected with Fer-1 and dipyridamole once a day. (n = 12 mice per group)

**k,** Kaplan-Meier survival curves of the WT and SLC7A11 KO mice treated with Fer-1 (2 mg kg^-1^) or dipyridamole (5 mg kg^-1^), followed by Dox (20 mg kg^-1^), then injected with Fer-1 and dipyridamole once a day. (n = 6 mice per group)

Data and error bars are mean ± SEM, n = 9 mice in **c** and **d**; n=5 (sham+vehicle), 4 (Dox +vehicle), 4 (Dox + dipyridamole) and 4 (Dox+Fer-1) mice in **e-g** and **i.** All *P* values were calculated using a two-tailed, unpaired Student’s t-test. Log-rank (Mantel-Cox) tests are used to compare the Kaplan-Meier survival curves of mice in **j** and **k**.


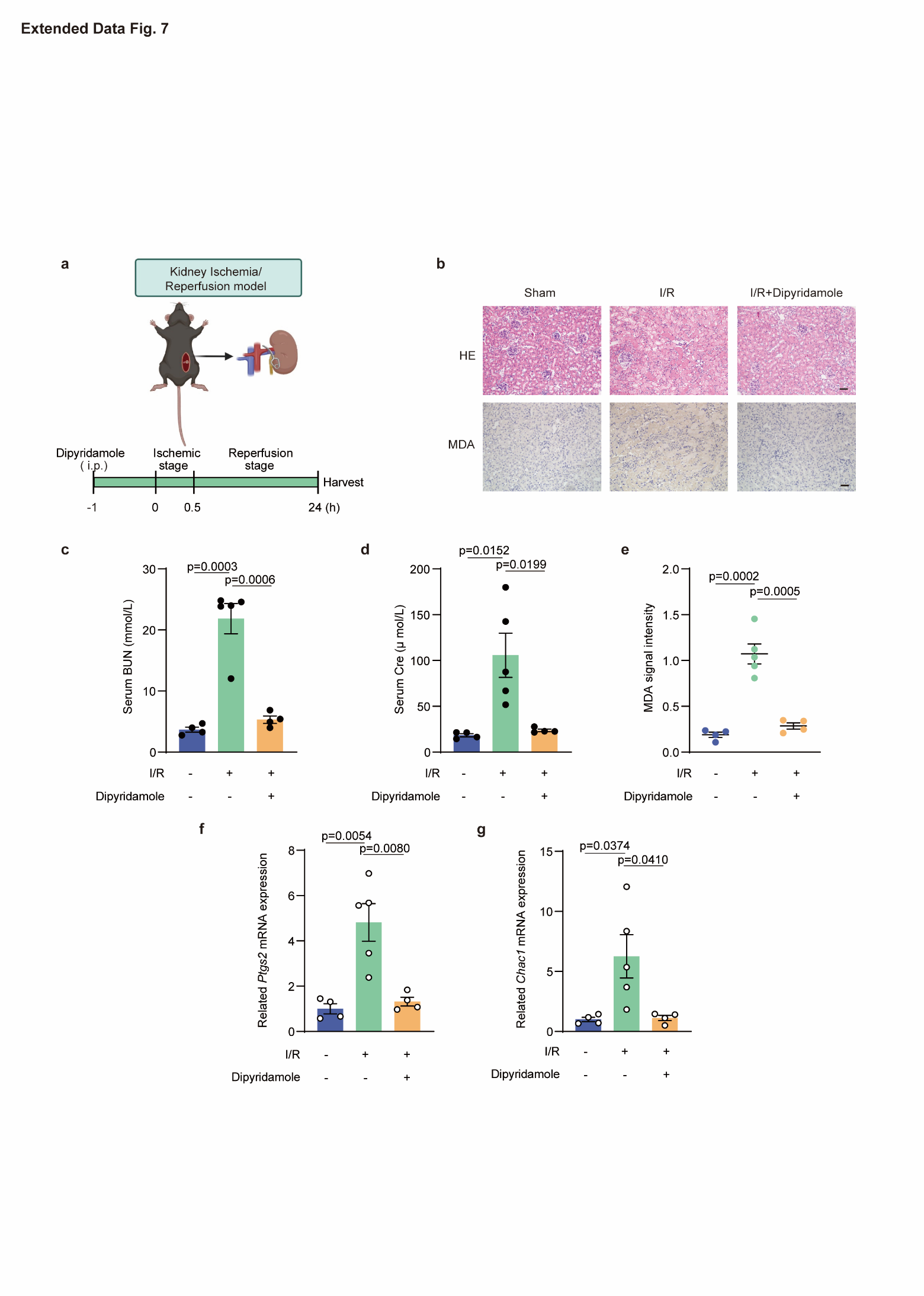


**Extended Data Fig. 7. Dipyridamole prevents against I/R-induced kidney damage.**

**a,** Pattern diagram of kidney I/R in mice. WT mice were intraperitoneally injected with vehicle or dipyridamole (10 mg kg^-1^) for 1 h, followed by 30 min of ischemia and 24 h for reperfusion.

**b,** Representative images of kidney HE staining and IHC staining of MDA in the indicated groups. Scale bars, 50 µm. Figure represents a total of n = 4 (sham+vehicle), 5 (I/R +vehicle) and 4 (I/R + dipyridamole) mice per group.

**c,d,** Serum BUN and Cre were measured in the indicated groups from (**a**). The number of mice per group in this figure is consistent with the figure (**b**) above.

**e,** MDA intensity was scored in the indicated groups from (**b**). (×100).The number of mice per group in this figure is consistent with the figure (**b**) above.

**f,g,** The relative mRNA levels of *Ptgs2* (**f**) and *Chac1* (**g**) were quantified by qRT-PCR in the indicated groups from (**a**). The number of mice per group in this figure is consistent with the figure (**b**) above.

Data and error bars are mean ± SEM, n = 4 (sham+vehicle), 5 (I/R +vehicle) and 4 (I/R + dipyridamole) mice in **c**-**g**. All *P* values were calculated using two-tailed, unpaired Student’s t-test.


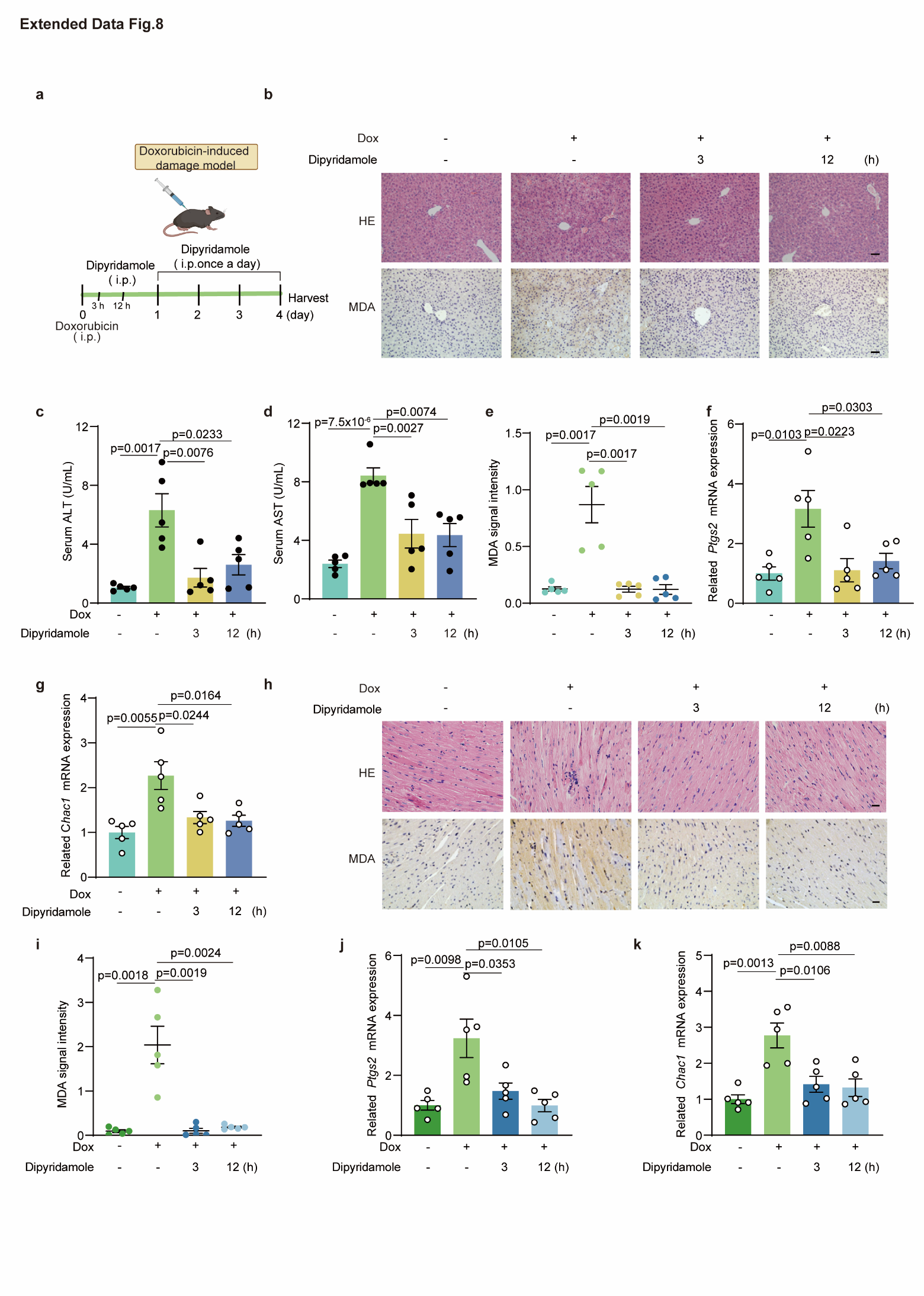


**Extended Data Fig.8. Dipyridamole prevents against Dox-induced organ damage.**

**a,** Pattern diagram of Dox-induced organ damage in mice. WT mice were pretreated with vehicle and Dox (20 mg kg^-1^) on day 0, then injected with vehicle and dipyridamole at indicated times once a day.

**b,** Representative images of liver HE staining and IHC staining of MDA in the indicated groups from (**a**). Scale bars, 50 µm. (n=5 mice per group)

**c,d,** Serum ALT and AST measurements in the indicated groups from (**a**).(n=5 mice per group)

**e,** MDA intensity was scored in the indicated groups from (**b**). (×100, n=5 mice per group)

**f,g,** The relative mRNA levels of *Ptgs2* (**f**) and *Chac1* (**g**) were quantified by qRT-PCR in mice livers in the indicated groups from (**a**). (n=5 mice per group)

**h,** Representative images of heart HE staining and IHC staining of MDA in the indicated groups from (**a**). Scale bars, 20 µm. (n=5 mice per group)

**i,** MDA intensity was scored in the indicated groups from (**h**). (×100, n=5 mice per group)

**j,k,** The relative mRNA levels of *Ptgs2* (**j**) and *Chac1* (**k**) were quantified by qRT-PCR in mice hearts in the indicated groups from (**a**). (n=5 mice per group)

Data and error bars are mean ± SEM, n = 5 mice in **c**-**g** and **i**-**k**. All *P* values were calculated using two-tailed, unpaired Student’s t-test.
